# Multi-omics analysis of the bioactive constituents biosynthesis of glandular trichome in *Perilla frutescens*

**DOI:** 10.1186/s12870-021-03069-4

**Published:** 2021-06-18

**Authors:** Peina Zhou, Mengjiao Yin, Shilin Dai, Ke Bao, Chenglin Song, Chanchan Liu, Qinan Wu

**Affiliations:** 1 College of Pharmacy, Nanjing University of Chinese Medicine, Nanjing, 210023 China; 2 Collaborative Innovation Center of Chinese Medicinal Resources Industrialization, Nanjing, 210023 China; 3 National and Local Collaborative Engineering Center of Chinese Medicinal Resources Industrialization and Formulae Innovative Medicine, Nanjing, 210023 China

**Keywords:** *Perilla frutescens* (L.) Britt, Glandular trichomes, Metabolite biosynthesis, Transcriptome

## Abstract

**Background:**

*Perilla frutescens* (L.) Britt is a medicinal and edible plant widely cultivated in Asia. Terpenoids, flavonoids and phenolic acids are the primary source of medicinal ingredients. Glandular trichomes with multicellular structures are known as biochemical cell factories which synthesized specialized metabolites. However, there is currently limited information regarding the site and mechanism of biosynthesis of these constituents in *P. frutescens*. Herein, we studied morphological features of glandular trichomes, metabolic profiling and transcriptomes through different tissues.

**Results:**

Observation of light microscopy and scanning electron microscopy indicated the presence of three distinct glandular trichome types based on their morphological features: peltate, capitate, and digitiform glandular trichomes. The oil of peltate glandular trichomes, collected by custom-made micropipettes and analyzed by LC–MS and GC–MS, contained perillaketone, isoegomaketone, and egomaketone as the major constituents which are consistent with the components of leaves. Metabolomics and transcriptomics were applied to explore the bioactive constituent biosynthesis in the leaves, stem, and root of *P. frutescens.* Transcriptome sequencing profiles revealed differential regulation of genes related to terpenoids, flavonoids, and phenylpropanoid biosynthesis, respectively with most genes expressed highly in leaves. The genes affecting the development of trichomes were preliminarily predicted and discussed.

**Conclusions:**

The current study established the morphological and chemical characteristics of glandular trichome types of *P. frutescens* implying the bioactive constituents were mainly synthesized in peltate glandular trichomes. The genes related to bioactive constituents biosynthesis were explored via transcriptomes, which provided the basis for unraveling the biosynthesis of bioactive constituents in this popular medicinal plant.

**Supplementary Information:**

The online version contains supplementary material available at 10.1186/s12870-021-03069-4.

## Background

In the plant kingdom, trichomes are specialized tissues from epidermal cells with diverse structures and functions that play essential roles in resistance to stress, UV radiation, secretion, and accumulation of special compounds [1, 2]. One type of trichomes, glandular trichomes (GTs), can synthesize, store, or secrete various bioactive metabolites, such as terpenoids, phenylpropanoids, flavonoids, alkaloids, and acyl sugars, and are therefore known as biochemical cell factories [3]. As a result, GTs are the main target for studying the synthesis and regulation of secondary metabolites in plants. The study of GTs in medicinal plants has attracted the interest of scientists, such as the GTs of *Schizonepeta tenuifolia, Mentha* x *piperita*, and *Artemisia annua*. Their GTs synthesize and store essential oils, which are widely used in fragrance industries, pharmaceuticals, and cosmetic products [4–6].

*Perilla frutescens* (L.) Britt is an edible vegetable and medicinal plant processed in cosmetics such as skin creams, soaps, and dermatological medicinal preparations [7]. Two kinds of trichomes are present on *P. frutescens* leaves, flowers, and stems with multicellular structures: GTs (peltate glandular trichomes PGTs and capitate glandular trichomes CGTs) and non-glandular trichomes (NGTs) [8]. Previous reports have noted that the GTs in *P. frutescens* were considered the primary tissue for essential oil synthesis and accumulation, especially PGTs. The number of essential oil components synthesized in leaves was positively correlated with the number of PGTs [9, 10]. The [^14^C]-sucrose tracer experiments showed that the monoterpenoid and phenylpropanoid components of the essential oil were synthesized by PGTs [9]. Histochemical localization further indicated that terpenoids, the main ingredients of essential oil, were present in PGTs and not CGTs [10]. Moreover, the metabolite profiles of PGTs in *P. frutescens* have not been reported. In addition to essential oil, there are a variety of other pharmaceutically bioactive secondary metabolites that are synthesized and accumulated in *P. frutescens,* such as phenolic acids (caffeic acid and rosmarinic acid), flavonoids, and volatiles (monoterpenes and sesquiterpenes), which give the plant pleasant green and purple colors, unique aromas, and medicinal uses [11]. Several studies have focused on secondary metabolite profiling of different tissues of *P. frutescens*, but only a few studies have examined their biosynthetic pathways, especially monoterpenes [12].

In the present study, we established the morphological and chemical characteristics of PGTs on the leaves of *P. frutescens*. Metabolomics and transcriptome databases from different tissues were used to identify candidate genes involved in the biosynthesis of bioactive constituents, and the genes affecting the development of trichomes were preliminarily predicted. These results enhanced our understanding of the mechanisms underlying the biosynthesis of bioactive constituents in *P. frutescens* and laid a solid foundation for the future investigation of trichomes, improvement of its health functions, and better-quality medicial plants.

## Results

### Three types of glandular trichomes are present on aerial surfaces on leaves

**Fig. 1:**
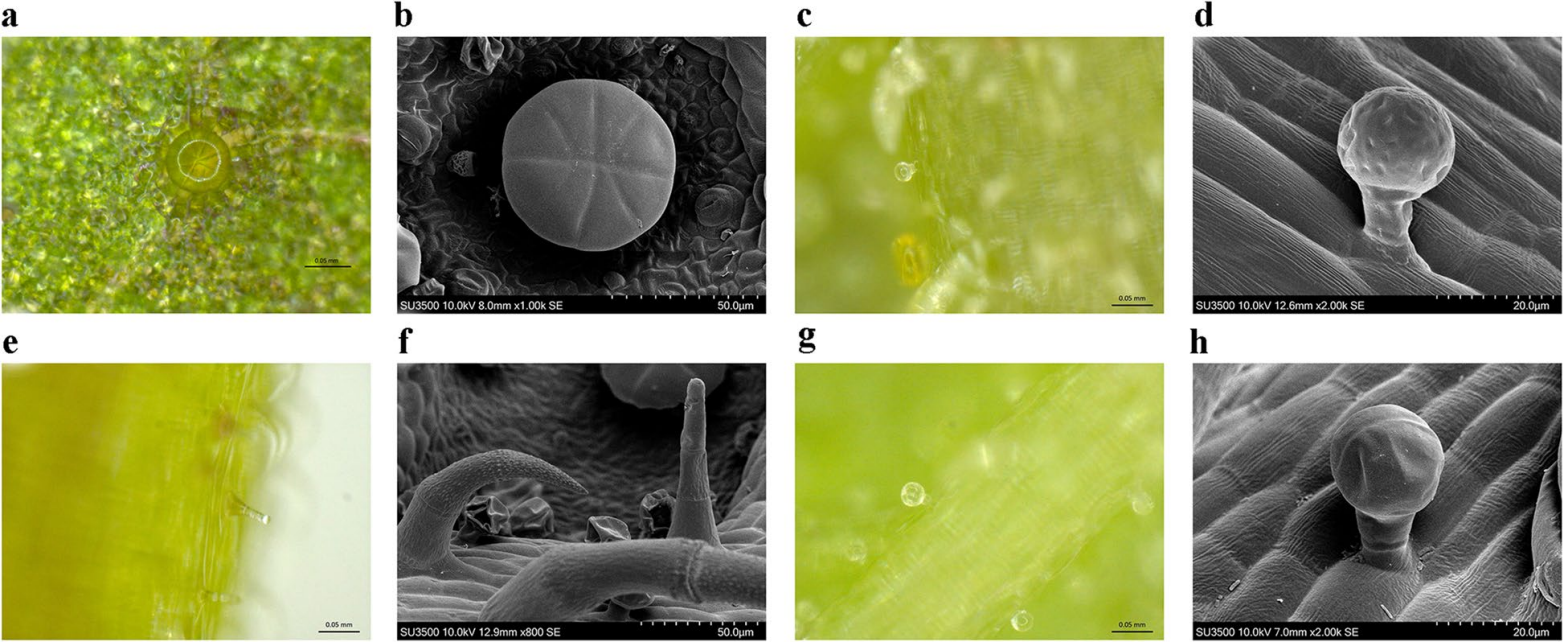
The morphology of glandular trichomes in *P. frutescens*; stereomicroscope and SEM for PGT (**a, b**), CGT (**c, d** for one head cell; **g, h** for two head cells) to DGT (**e, f**) (bar = 0.05 mm and 0.02 μm for **d, h**)

The whole plant of *P. frutescens* is densely pubescent. Its leaves are green and soft purple on the adaxial and abaxial surfaces, respectively, which is called the “double color *P. frutescens*.” The primary roots of *P. frutescens* are shorter than those of the lateral roots (Supplementary Fig. 1). The plants in our study were similar to the 465P and green/red *Perilla* in previous reports [11, 13]. The pubescence of *P. frutescens* was composed of multicellular GTs and NGTs [8]. The GTs were including PGTs, CGTs and another morphology GTs, with finger-like shape, named digitiform glandular trichomes (DGTs). These three type GTs all contained three parts including basal cells, stalk cells, and head cells, like GTs in other Lamiaceae plants [4, 5]. These three types of GTs located on leaves and stems (Fig. 1, Supplementary Fig. 1), which were captured using an SEM and a stereomicroscope. PGTs consisted of multicellular heads, one stalk cell, and one basal cell, which assumed a globular dome shape at maturity (Fig. 1 a, b). It should be noted that the secretions of PGTs are yellow when mature. PGTs were approximately 42–47 μm in height and 56–60 μm in diameter (with four and eight secretory cells) when mature. CGTs were smaller than PGTs, with a 23–28 μm diameter spherical head and 31–34 μm in height, characterized by a short stalk, one or two-celled head, and one basal cell (Fig. 1 c, d, g, h). DGTs consisted of one single head cell, a longer stalk cell, and one basal cell (Fig. 1 e, f). They were approximately 61–69 μm in height and 10–13 μm in diameter when mature (Supplementary Fig. 2 and 3).

### Metabolites of PGTs and leaves, stems, and roots of *P. frutescens*

**Fig. 2:**
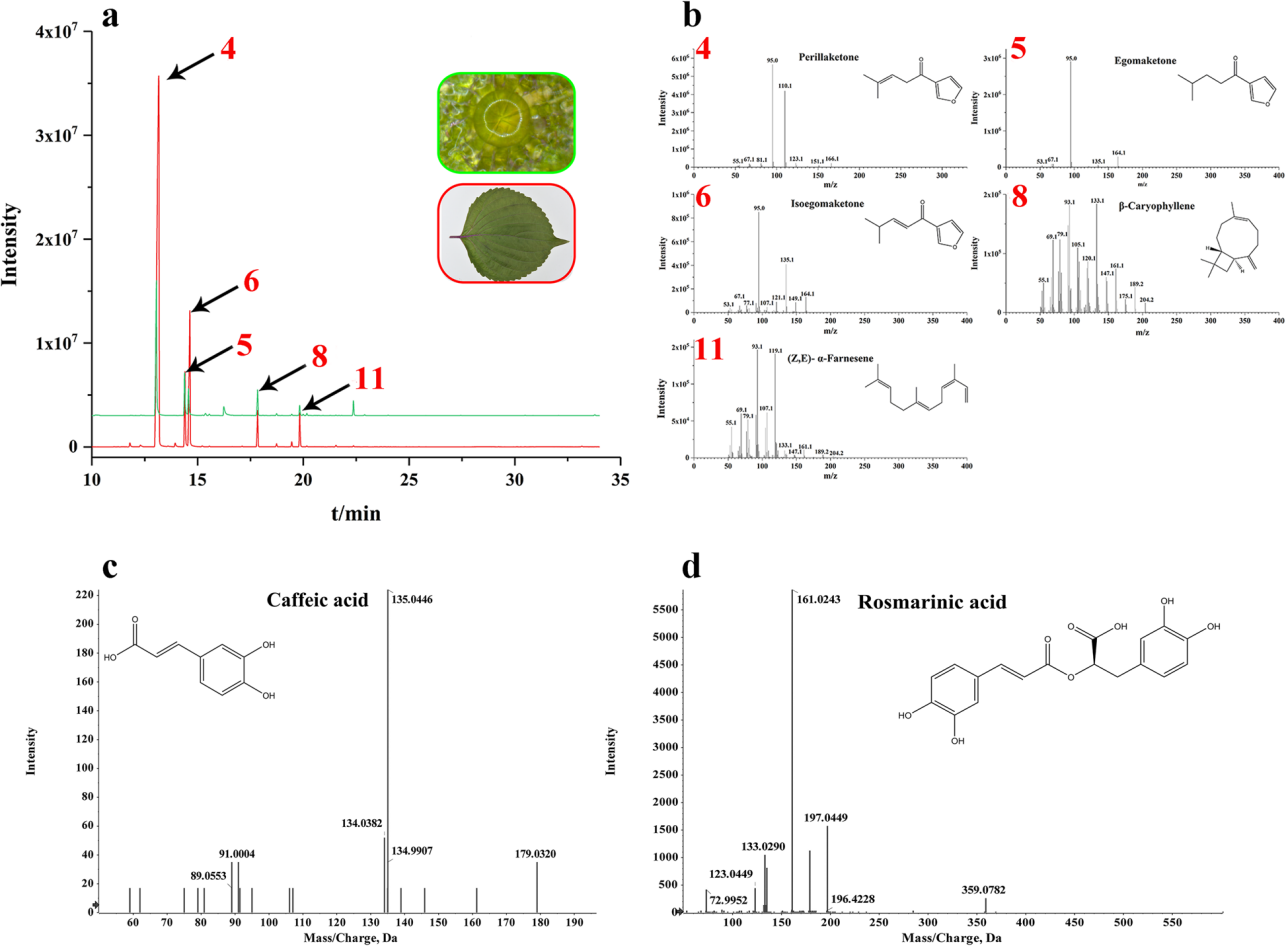
GC–MS peaks of leaves and PGTs (**a**), and the mass spectra of major compounds in GC–MS (**b**); the mass spectra of Caffeic acid (**c**) and Rosmarinic acid (**d**) in LC–MS

The components of volatile oil contributed the most to the aroma of *P. frutescens* and were the active ingredients in the drugs. Volatile oil was the primary evaluation criterion for the quality of medicinal materials in the *Chinese Pharmacopeia* (2015 edition). We measured leaves, stems, roots, and PGT volatiles in plants by GC–MS to investigate the volatile constituents. Essential oil was collected by SDE from leaves (enriched in PGTs), stems, and roots. Secretions from PGTs were collected using micropipettes diluted with solvent, and then analyzed by GC–MS. There were 16 peaks in PGTs, 19 peaks in leaves, 7 peaks in stems, and 7 peaks in roots. 13 peaks have been previously identified, and eight peaks were unknown. The results of the identified peaks are listed in Table 1, and most of the peaks appeared 10 min later. GC–MS peak mass spectra for the compounds are shown in Supplementary Fig. 4. Based on the GC–MS results, the oil composition of PGTs was similar to that of distillates from leaves (Fig. 2a), implying that these PGTs were the dominant source of commercial essential oils. Among these compounds, terpenoids were shown as the leading group. Perillaketone, the main compound, accounts for approximately 50% of the volatile oil of leaves, stems, and PGTs, and the isoegomaketone content in leaves was close to 20%, which implied that the chemical type of *P. frutescens* is PK-II [14]. Moreover, the volatile composition of PGTs, which are known to be the primary tissues involved in the biosynthesis or accumulation of volatile oil, was strikingly similar to that of the leaves, which shared 11 common identified compounds. The mass spectra of the primary compounds in GC–MS are shown in Fig. 2b, containing perillaketone (4), egomaketone (5), isoegomaketone (6), β-caryophyllene (8), and (Z,E)-α-farnesene (11).

**Table 1:** The compounds identified in the essential oils of *P. frutescens*

No	Compund name	Retention time (min)	Formula	Molecular Mass (g/mol)	Type	Relative content (%)			
						**Glandular trichome**	**Leaf**	**Stem**	**Root**
1	(Z)-3-Hexenol	4.23	C_6_H_12_O	100.16	Alcohol	N	0.71	N	N
2	1-Octen-3-ol	6.65	C_8_H_16_O	128.21	Alcohol	N	1.09	1.10	6.32
3	Linalool	8.89	C_10_H_18_O	154.25	Monoterpenoid	1.33	0.93	3.03	N
4	Perillaketone	13.05	C_10_H_14_O_2_	166.22	Monoterpenoid	48.65	65.31	68.32	3.47
5	Egomaketone	14.38	C_10_H_12_O_2_	164.2	Monoterpenoid	15.26	6.03	N	N
6	Isoegomaketone	14.57	C_10_H_12_O_2_	164.2	Monoterpenoid	9.96	15.62	N	N
7	Elixene	15.56	C_15_H_24_	204.35	Sesquiterpene	0.41	0.09	N	N
8	β-Caryophyllene	17.84	C_15_H_24_	204.35	Sesquiterpene	7.16	3.48	7.20	1.95
9	α-Humulene	18.74	C_15_H_24_	204.35	Sesquiterpene	0.56	0.3	N	N
10	Germacrene D	19.45	C_15_H_24_	204.35	Sesquiterpene	0.41	0.51	1.65	N
11	(Z,E)-α-Farnesene	19.83	C_15_H_24_	204.35	Sesquiterpene	2.68	3.37	5.25	N
12	α-Farnesene	20.16	C_15_H_24_	204.35	Sesquiterpene	0.63	0.09	N	N
13	(6E)-Nerolidol	21.54	C_15_H_24_	204.35	Sesquiterpene	0.12	0.14	N	N

N represented not detected

To comprehensively identify the metabolite profiles of *P. frutescens*, the compounds were authenticated using LC–MS, which was dedicated to the *P. frutescens* color and medicinal usage. Therefore, metabolite profiles in the four organs were tentatively investigated by UPLC-ESI-Q-TOF–MS/MS through the full scan negative and positive ESI modes. In our study, the compositions of different organs showed significant differences. Supplementary Table S1 shows the retention times, molecular formula, and mass spectral data (molecular and fragment ions) of the identified metabolites. LC–MS peaks and mass spectra of the compounds are shown in Supplementary Fig. 5. We identified 71 compounds from *P. frutescens* through negative and positive modes, of which flavonoids (16), phenylpropanoids (15), and terpenoids (13) accounted for a large proportion. There were 36, 63, 46, and 24 compounds identified in the PGTs, leaves, stems, and roots, respectively. Except for roots, the top three compounds in the other parts were flavonoids, phenylpropanoids, and terpenoids. Caffeic acid and rosmarinic acid were detected as the predominant metabolites [15]. Interestingly, similar to the results of GC–MS, the compounds accumulated in PGTs were strikingly similar to leaves, which shared 30 common compounds.

The fragmentation behaviors of the above two compounds are shown as examples. The product ion mass spectrum of compound 10 (t_R_ = 3.2 min) showed a deprotonated ion [M-H]^−^ at m/z 179.0320. In the MS/MS spectrum, the distinctive product ion at m/z 135.0446 ([M-H]^−^-44 amu) corresponded to the loss of a -COOH moiety, which was confirmed to be caffeic acid (10), with the elemental formula C_9_H_8_O_4_ (Fig. 2c) [16]. Compound 27 (t_R_ = 4.85 min) exhibited a deprotonated molecular ion at m/z 359.0772. The MS/MS fragmentation patterns possessed three fragment ions at m/z 197.0455, 179.0360, and 161.0243. The fragment ion at m/z 197.0455 was formed by the loss of m/z 162 fragment (m/z 359–m/z 197; [M-H]^−^-162 amu), which was determined to be the characteristic of a caffeoyl moiety [17]. The fragment ion at m/z 161.0243 corresponded to the deprotonated caffeoyl residue. Thus, compound 27 was tentatively assigned to rosmarinic acid (27) (C_18_H_16_O_8_) (Fig. 2d). The identified caffeic acid and rosmarinic acid were confirmed using the retention time of the standard mixture (Supplementary Fig. 6 A, B). Perillaketone and isoegomaketone were also identified by LC–MS using a standard mixture (Supplementary Fig. 6 C, D).

### RNA-seq and de novo assembly and annotations result

Through Illumina high-throughput sequencing of different tissues of *P. frutescens*, 52.8, 41.0, and 42.2 billion clean reads were obtained from leaves, stems, and roots, respectively (Supplementary Table S2 and Table S3). The clean reads were assembled into contigs and de novo assembled into transcripts using Trinity, yielding a total of 315,005 transcripts and 117,939 unigenes (Supplementary Table S4). A total of 117,939 unigenes were annotated using common databases including the CDD, KOG, NR, NT, PFAM, SwissProt, TrEMBL, GO, and KEGG, to which approximately 34.43%, 28.8%, 43.94%, 31.45%, 25.02%, 42.73%, 43.23%, 46.03%, and 4.74% of unigenes were mapped, respectively (Supplementary Table S5). Through homologous species comparison in the NR database, 36.32% of the unigenes had the highest homology with *Sesamum indicum* (Pedaliaceae), followed by *Erythranthe guttata* (Scrophulariaceae) (Supplementary Fig. 7). The details of the GO and KEGG analyses are shown in Supplementary Fig. 8 A and B.

### Identification of DEGs and annotations result

A total of 10,696 DEGs were collected with the filter criteria |FoldChange|> 2 and q-value < 0.05, including 4127 upregulated genes and 4088 downregulated genes in L vs. R, 2548 upregulated and 2574 downregulated in L vs. S, and 3203 upregulated and 2898 downregulated in S vs. R, respectively (Supplementary Fig. 9 A). There were 1126 common  unigenes in three comparisons (Supplementary Fig. 9 B). DEGs of S vs. R, L vs. S, and L vs. R comparisons were assigned to GO classifications shown in Supplementary Fig. 10 A-C. The responses to stimuli, metabolic processes, and cellular processes included in the biological process were enriched in three comparisons; catalytic activity in molecular function was also enriched in three comparisons. To explore the molecular basis of trichome development among leaves, stems, and roots, a large number of unigenes were assigned to GO annotation (128, GO:0,010,026 trichome differentiation; 57, GO:0,010,091 trichome branching; 100, GO:0,010,090 trichome morphogenesis) (Supplementary Table S6). In the above unigenes, most were highly expressed in leaves or stems (Supplementary Fig. 11), which was consistent with the phenomenon that GTs accumulated at these organs. In the KEGG pathway analysis, pathways such as plant hormone signal transduction and biosynthesis of phenylpropanoids, flavonoids, monoterpenoids, terpenoid backbones, sesquiterpenoids, and triterpenoids were enriched in S vs. R and L vs. R. As for L vs. S, only phenylpropanoid, flavonoid, sesquiterpenoid, and triterpenoid biosynthesis were enriched, revealing the same trends as the distribution of secondary metabolites (Supplementary Fig. 10 D-F).

### DEGs related to secondary metabolites

**Fig. 3:**
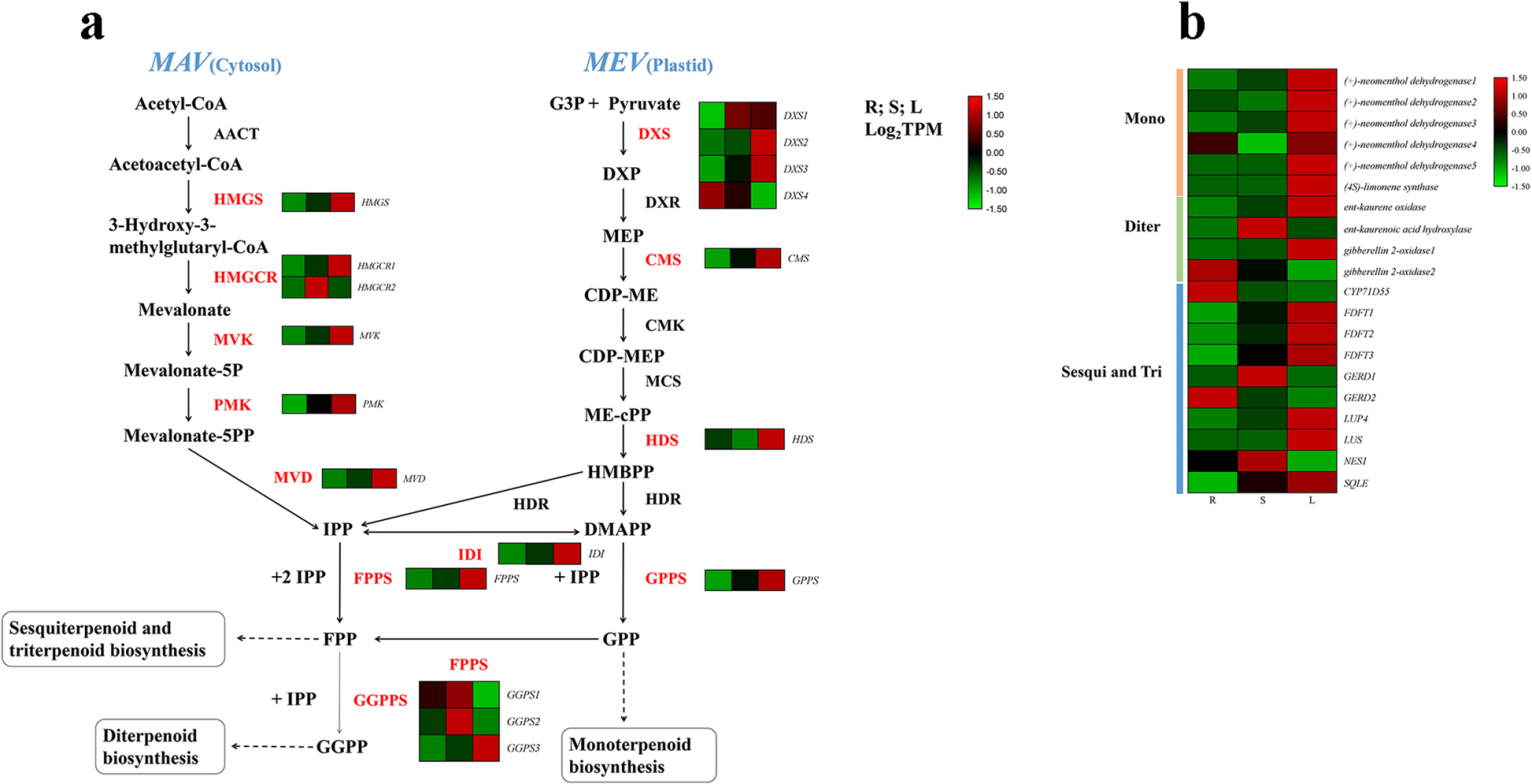
The biosynthesis of terpenoids (**a**) and heatmap (**b**) of DEGs involved in monoterpene, diterpenoid and sesquiterpene biosynthesis; *AACT,* acetyl-CoA acetyltransferase; *HMGS*, hydroxymethylglutaryl-CoA; *HMGCR*, hydroxymethylglutaryl-CoA reductase synthase; *MVK*, mevalonate kinase; *PMK*, phosphomevalonate kinase; *MVD*, mevalonate diphosphate decarboxylase; *DXS*, 1-deoxy-D-xylulose-5-phosphate synthase; *DXR*, 1-deoxy-D-xylulose-5-phosphate reductoisomerase; *CMS*, 2-C-methyl-D-erythritol 4-phosphate cytidylyltransferase; *CMK*, 4-diphosphocytidyl-2-C-methyl-D-erythritol kinase; *MCS*, 2-C-methyl-D-erythritol 2,4-cyclodiphosphate synthase; *HDS*, 4-hydroxy-3-methylbut-2-enyl diphosphate synthase; *HDR*, 4-hydroxy-3-methylbut-2-enyl diphosphate reductase; *IDI*, isopentenyl pyrophosphate isomerase; *GPPS*, geranyl diphosphate synthase; *FPPS*, farnesyl diphosphate synthase; *GGPPS*, geranylgeranyl diphosphate synthase; *CYP71D55*, premnaspirodiene oxygenase; *FDFT1*, farnesyl-diphosphate farnesyltransferase; *GERD*, (-)-germacrene D synthase; *LUP4*, beta-amyrin synthase; *LUS*, lupeol synthase; *NES1*, (3S,6E)-nerolidol synthase; *SQLE*, squalene monooxygenase. Unigenes levels data represent by TPM. The red letters represent DEGs. Red and green represent high and low expression levels, respectively

The pathways annotated in the KEGG database were involved in the biosynthesis of secondary metabolites. We combined the analysis of transcriptomic and metabolomic data to explore key genes involved in terpenoid, flavonoid, and phenylpropanoid biosynthesis in three tissues. The metabolism of the terpenoids and polyketides subcategory contained eight pathways, and the largest number of DEGs (23 unigenes) were mapped to terpenoid backbone biosynthesis (Supplementary Fig. 12). Corresponding to the metabolic profiles and terpenoid biosynthesis, we aimed at the biosynthesis of terpenoid backbones, monoterpenoids, diterpenoids, sesquiterpenoids, and triterpenoids. Among these unigenes, 38 DEGs were identified as encoding 24 key enzymes that controlled terpenoid biosynthesis, and the details are listed in Supplementary Table S7. These unigenes were mainly mapped in the 2-C-methyl-D-erythritol 4-phosphate and mevalonate pathways (Fig. 3). Of the four *DXS* genes, *DXS1*, *DXS2*, and *DXS4* showed the highest expression in leaves, and they were located upstream of terpenoid biosynthesis. These results suggest that *DXS* is important for the generation of 2-C-methyl-D-erythritol 4-phosphate pathways and deserves further functional characterization. The genes related to monoterpene biosynthesis also presented the same trends, which may lead to the accumulation of monoterpenes [9].

**Fig. 4:**
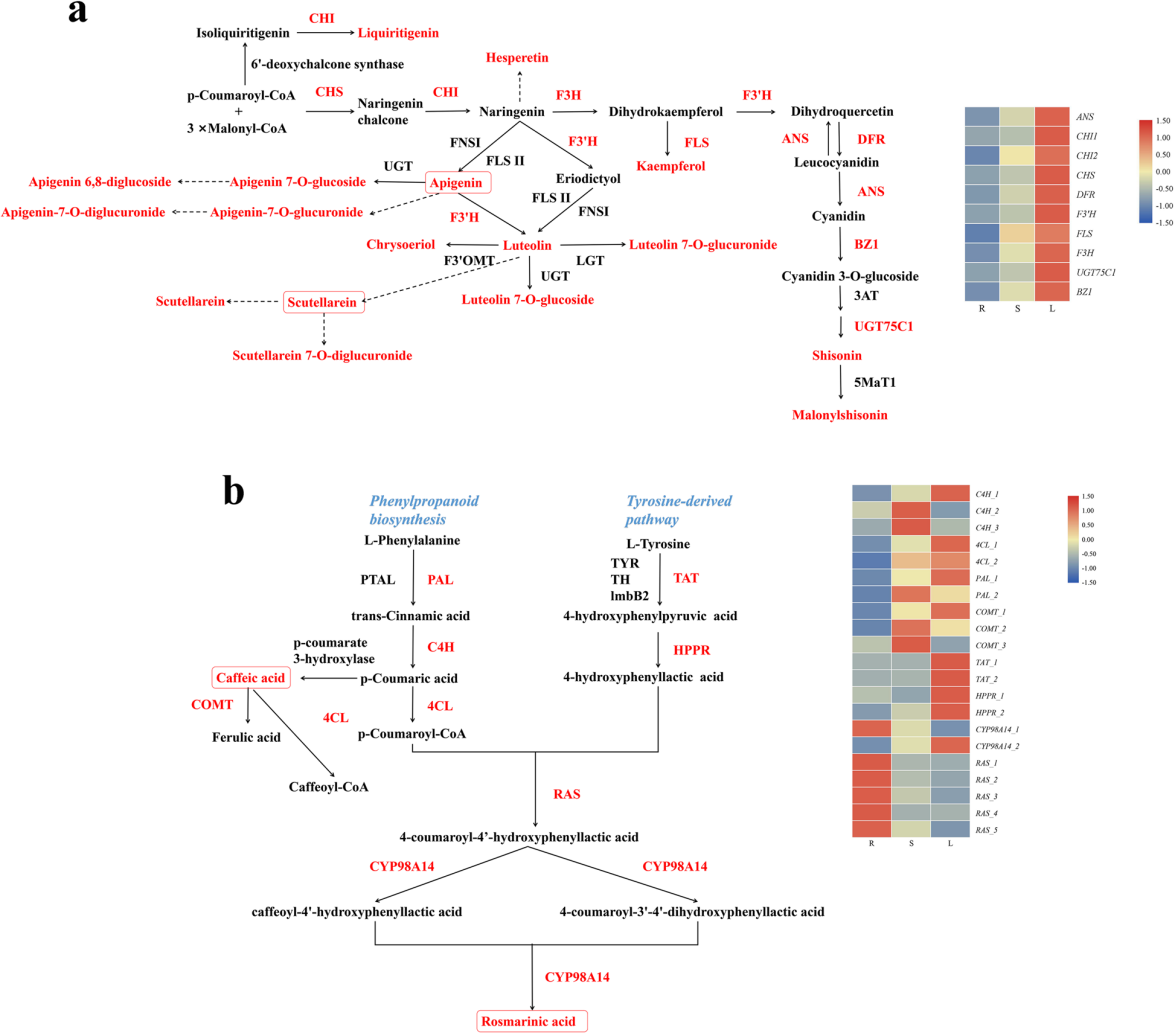
The biosynthesis of flavonoids (**a**) and phenylpropanoids (**b**) according KEGG. *PAL*, phenylalanine ammonia-lyase; *C4H*, trans-cinnamate 4-monooxygenase; *4CL*, 4-coumarate–CoA ligase; *COMT*, caffeic acid 3-O-methyltransferase; *TAT*, tyrosine aminotransferase; *HPPR*, hydroxypyruvate reductase; *RAS*, rosmarinate synthase.Unigenes levels data represent by TPM. The red letters represent DEGs and detected compounds. Red and blue represent high and low expression levels, respectively

The leaves and stems of *P. frutescens* showed pleasant purple and green colors, which were genetically controlled to accumulate a significant amount of anthocyanin (especially malonylshisonin) [18]. In our study, 10 DEGs were related to flavonoid biosynthesis and upstream of anthocyanin biosynthesis, which were all highly expressed in leaves (Fig. 4a). Combined with phenylpropanoid biosynthesis and tyrosine-derived pathways, 21 unigenes encoding eight key genes were assigned to rosmarinic acid and caffeic acid biosynthesis (Fig. 4b) [19]. DEGs in the above two pathways showed similar expression patterns, except that *RASs* were highly upregulated in roots. Metabolites and other DEGs involved in phenylpropanoid biosynthesis are listed in Supplementary Fig. 13.

### DEG related to trichome development

**Fig. 5:**
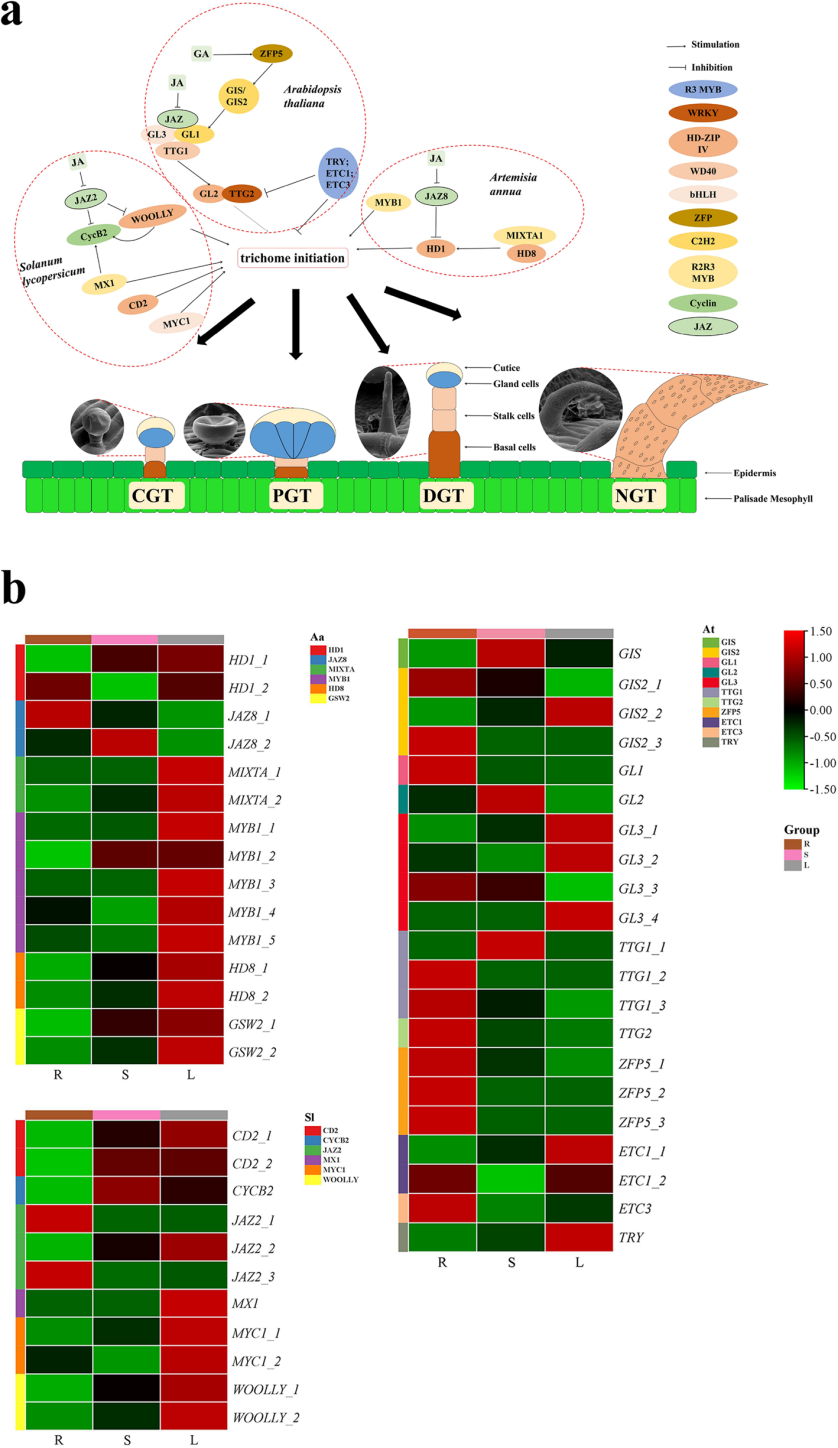
A map of genes regulation for the growth (**a**) and development of trichomes. Heatmap of genes related to trichomes growth and development (**b**). Unigenes levels data represent by TPM. Red and green represent high and low expression levels, respectively

Trichomes are classified as non-glandular or glandular. *Arabidopsis thaliana* has single cell non-trichomes, whereas *Solanum lycopersicum* and *Artemisia annua* have multicellular glandular trichomes. The genes related to trichome initiation and development have been widely reported, including *TTG1*, *GL1*, and *GL3* of *A. thaliana; MYC1*, *MX1*, and *CD2* of *S. lycopersicum*; and *MIXTA*, *MYB1*, and *HD1* of *A. annua.* According to these reports, 46 unigenes, encoding 21 key genes, were screened in our transcriptomes (Fig. 5). Except for the 21 homologous unigenes of *A. thaliana*, the expression patterns of homologous genes of *A. annua* (13 unigenes) and tomato (12 unigenes) were similar. The homologous genes like *SlMYC1*, *SlMX1*, *SlCD2*, *SlWOOLLY, AaMIXTA*, *AaMYB1*, *AaHD1*, *AaHD8*, and *AaGWS2*, which have been reported to promote trichomes development, all expressed highly in leaves. Among the 46 trichome development-related unigenes, most were TFs belonging to R2R3 MYB, WD40, bHLH, and HD-ZIP IV family.

Plant hormones have also been reported to regulate trichome initiation and development, such as gibberellin (GA) and jasmonic acid (JA) [6, 20]. Our transcriptome data analysis revealed that multiple genes were mapped to activated signaling pathways. In the GA and JA pathways, we identified 24 and 37 unigenes, respectively (Supplementary Fig. 14). Additionally, 16 *DELLA* and 18 *JAZ* were screened among these unigenes, which negatively regulated the growth and development of trichomes [21, 22]. As expected, most *DELLA* and *JAZ* were downregulated in leaves enriched with trichomes.

### Gene expression validated by qRT-PCR

**Fig. 6:**
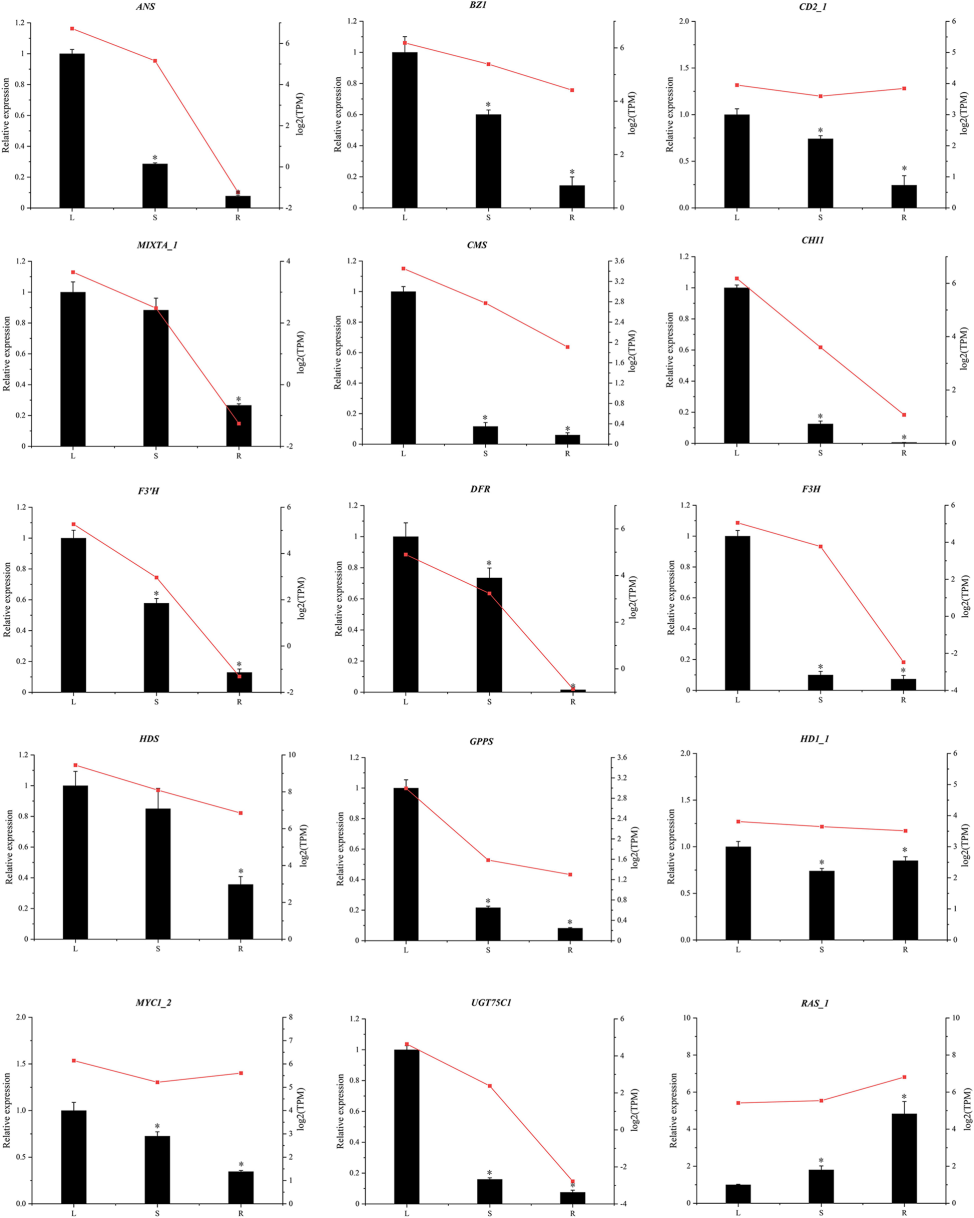
Validation of expression patterns of 15 DEGs by qRT-PCR. The relative expression levels were calculated according to the 2^-ΔΔCT^ method using β-actin as internal reference gene. Error bars represented standard error of mean. * indicated significant difference of the expression means at p < 0.05 between L and S or L and R (n = 4). The relative expression of qRT-PCR was indicated on the left y-axis and the TPM normalized expression level (log2_TPM_) of RNA sequencing was indicated on the right y-axis

Fifteen DEGs were selected to verify the accuracy of the transcriptome data using qRT-PCR. These key structural genes are involved in the biosynthetic pathways of terpenoids, flavonoids, and phenolic acids, and trichome formation. Specific primers were designed using Primer Premier 5.0 (Supplementary Table S8). In general, the expression patterns determined by qRT-PCR showed similar trends to RNA-Seq  (Fig. 6 and Supplementary Table S9), which confirmed the accuracy of the RNA-Seq results reported in this study.

## Discussion

*P.frutescens* is a well-known medicinal and edible plant that has been widely cultivated in Asia more than 2000 years [23]. The leaves and stems often present green or purple colors. Volatile oil is one of the main components of *P. frutescens*, and different chemotypes have been described based on the main components of essential oils [24, 25]. The chemotypes are listed in Supplementary Table S10, and their speculated synthesis pathways are shown in Supplementary Fig. 15 [14]. *P. frutescens* in our study was identified as a PK-II type cultivated widely in China. To further mine nutraceutical and medicinal potential, we integrated metabolite profiling and transcriptome analysis of different tissues. Additionally, we displayed the metabolic profiles of PGT secretion for the first time.

### Morphological and distribution of GTs in P. frutescens

In previous reports, it has been elaborated that *P. frutescens* has two types of glandular trichomes (PGTs and CGTs), and PGTs were the primary tissues of biosynthesis and accumulation of essential oils with a predominant number and size [9, 10]. We certified another finger-like shaped GT, named DGT (Fig. 1 and Supplementary Fig. 2 I-L), similar to the type II CGTs with a unicellular base, two elongated stalk cells, and one head cell [10]. The number of secretory cells was consistent with published papers (PGT usually with 8 cells, CGTs usually with one or two cells) [8–10]. Under a stereomicroscope, the PGTs were filled with yellow essential oil in a large sub-cuticular storage space formed by the separation of the cuticle from secretory cells, such as PGTs in peppermint [26]. Compared with PGTs, the storage capacity of CGTs was limited to smaller sizes and fewer secretory cells. As shown in Supplementary Fig. 2E-G, there were two types of CGTs containing colored and transparent glandular heads. Whether there are two types of CGTs, or their developmental sequence, needs to be further studied. As for DGTs, there was only one transparent head cell that was unknown to have a secretory capacity. Its base cell may enlarge during development, as shown in Supplementary Fig. 2 I-K. PGTs, CGTs and DGTs were distributed on both the adaxial and abaxial leaf (mostly) surface and stems and were especially abundant in the central vein and the area near the leaf base. In summary, the morphological features and occurrence of GTs in our data were similar to previous reports of *P. frutescens*, which is also similar to GTs in *S. tenuifolia* [5] and *Mentha* x *piperita* [27]*,* belonging to Labiaceae.

### Metabolites variation of PGTs, leaves, stems, and roots

Metabolite profiling of PGTs, leaves, stems, and roots showed that the most enriched compounds were terpenoids, flavonoids, phenolic acids, and fatty acids (Table 1, Supplementary Table S1), and the confirmed compounds in PGTs were strikingly similar in leaves. Theoretically, the compounds contained in PGTs should be detected in leaves, whereas the leaf extracts are more abundant than PGT secretions. In addition to PGTs, the leaves contained other types of cells and GTs, and the energy and heat generated in the process of ultrasonic extraction resulted in changes or loss of compounds. The method, which used micropipettes to collect secretions from PGTs, resulted in metabolic profiles closer to those of compounds in vivo. Egomaketone was the precursor of perillaketone and isoegomaketone (Supplementary Fig. 15). The contents of perillaketone and isoegomaketone were lower in PGTs than in leaves. In contrast, the egomaketone content was the opposite because the leaves in node 1 (from the tip of the stem to the root) for PGT analysis were younger than leaf extraction (nodes 1 and 2), which may reveal the developmental dynamics of leaf essential oil composition. The relative contents of monoterpenoids and sesquiterpenoids in GC–MS were very low or absent in the roots. In the LC–MS analysis of *P. frutescens*, phenolic acid and flavonoids were generated from flavonoid and phenylpropanoid biosynthesis, respectively [15]. The purple leaves enriched anthocyanin pigments, such as malonylshisonin [18, 28]. It was detected in PGTs and leaves with the highest intensity in PGTs compared with other tissues. Interestingly, PGTs with higher intensity were gold, while the abaxial surface of the leaves was purple. Anthocyanin is sensitive to light, temperature, and pH [29]. These analyses confirmed that PGTs contained the bulk of the bioactive constituents accumulated by *P. frutescens*.

### Molecular mechanism of metabolites biosynthesis

Monoterpenes and sesquiterpenes are the main components of the volatile oil in *P. frutescens*. Crossing experiments between the principal types of *Perilla* suggested that the different chemotypes were under strict genetic control [30]. The main components of volatile oil in these chemotypes were generated from the mevalonic and shikimic acid pathways (Supplementary Fig. 15). The presence of the *G* gene is essential for the initiation of monoterpenoid biosynthesis via the mevalonic acid pathway. When the initiation was controlled by the recessive gene *h*, tans-citral was catalyzed by geranyl pyrophosphate, and under the regulation of multiple genes, perillaketone was finally generated [30]. Although the biosynthetic pathways for essential oils in *Perilla* have been described, the key genes controlling these pathways are still unknown. However, the lack of the *Perilla* genome made it a great challenge to unravel the complete biosynthesis pathway of monoterpenes in *P. frutescens*. A large number of unannotated unigenes (44.03%, Supplementary Table S5) in our transcriptome needed further exploration, which may be annotated as the key genes for the synthesis of perillaketone in the future.

The key gene for anthocyanidin, *ANS*, is located in the epidermal cells of the stems, where anthocyanins accumulate [28]. The stem transverse section in Supplementary Fig. 16 shows similar results. *ANS*, *DFR*, *BZ1*, and *UGT75C1* were the key genes in the biosynthetic pathway of malonylshisonin, and these DEGs expressed highly in leaves were consistent with anthocyanin intensity in LC–MS data. The biosynthesis of rosmarinic acid is generated from both the general phenylpropanoid pathway and a tyrosine-derived pathway [19]. The *RAS* coupled products from the two pathways and then hydroxylated by *CYP98A14* to form rosmarinic acid (Fig. 4) [31]. Seven DEGs were annotated as *RAS* (5) and *CYP98A14* (2), and most of them were highly expressed in roots with the lowest rosmarinic acid intensity. This observation may be attributed to the possible involvement of the predicted genes in other metabolic pathways in the roots.

### Genes and plant hormones related to trichome development

Compared with the transcriptomes of leaves with dense trichomes and roots without trichomes, the genes related to trichome initiation and development could be preliminarily analyzed by qRT-PCR. Some homologous genes related to trichome initiation and development were selected from our data (Fig. 6). In *A. thaliana*, TTG1, GL3, and GL1 consist of the WD40/bHLH/MYB complex, which regulates downstream *GL2* and *TTG2* expression to stimulate the differentiation of epidermal cells into non-trichomes [1, 32]. In *A. annua*, *AaHD8* interacts with *AaMIXTA1* and regulates the expression of *AaHD1* to induce GT initiation [33]. *SlMX1,* *SlCD2*, the closest homologs of *AaHD8*, *WOOLLY*, *SlMYC1*, and *SlCycB2*, positively regulate the initiation of GTs [1, 34, 35]. The expression patterns of homologous genes of *A. annua* and tomato were similar, and most genes were highly expressed in the leaves. However, the expression of homologous genes in *A. thaliana* was entirely different. The functions of these homologous genes will be further studied in *P. frutescens*. Due to their diverse trichome types, the trichome in *A. thaliana* was a single-celled NGT, while *A. annua* and tomato have multicellular GTs, which are similar to GTs in *P. frutescens* (Fig. 5), suggesting that the gene regulatory network of GTs formation was different from NGTs formation in *A. thaliana.* However, several genes may have a conserved function in the regulation of trichome initiation in *A. thaliana, A. annua,* and tomato [2, 32, 36, 37]. For example, *AtMYB61* in *A. thaliana* was the ortholog of *A. annua AaMYB1*, and myb61 mutants showed lower NGT density, suggesting that *AtMYB61* positively regulated NGTs initiation in *A. thaliana* [2, 32, 36, 37]*; SlTRY* was the ortholog of *AtTRY,* an inhibitor of NGT initiation in *A. thaliana*. When transforming *SlTRY* into *A. thaliana*, the development of NGTs was significantly inhibited [36].

In addition, GA and JA hormones regulated trichome development through the transcriptional regulation of *ZFP* and *JAZ*, respectively (Supplementary Fig. 14). Although trichome types in the above three kinds of plants were diverse, the hormone regulation of trichome development shared similar patterns. Thus, in the plant kingdom, plant hormones may share a common regulatory network. Besides regulating trichomes, JA and GA can increase the synthesis of secondary metabolites, such as terpenoids. JAZ and DELLA can be degraded by exogenous application of JA and GA, then the transcriptional activity of *MYC2* was released, which promoted the expression of *TPS* and increased the synthetic rate of terpenoids [38]. *JAR1* and *MYC2*, known as activated transcription factors in the JA signaling pathway, were highly expressed in the leaves. Their high expression can increase the content of phenylpropanoids, terpenoids, and alkaloids, which played an essential role in regulating stress signal transduction in plants [39, 40]. Thus, cultivating densely spaced GTs in *P. frutescens* with high medicinal and nutritional qualities is a future target. There will be great interest in investigating the molecular basis of *P. frutescens* trichome development and the complete characterization of monoterpene biosynthesis.

## Conclusion

In this study, we presented conclusive evidence for metabolic profiling of PGT-accumulated terpenoids (perillaketone, isoegomaketone, and egomaketone) and the transcriptome and metabolite profiling of leaves, stems, and roots revealing biosynthesis of terpenoids, flavonoids (malonylshisonin), phenolic acids (rosmarinic acid and caffeic acid). Genes, such as *DER* and *ANS*, related to malonylshisonin biosynthesis were highly expressed in leaves with a higher intensity of malonylshisonin. The genes belonging to the R2R2-MYB and HD-ZIP IV family, such as *MIXTA*-like *and HD1*-like, may regulate trichome development in *P. frutescens*. This study provided new insights into the morphology, metabolic profiles, and biosynthesis pathways of the main compounds. In the future, further research is required to uncover a novel molecular mechanism of the regulatory networks involved in the formation of trichomes and monoterpenoid biosynthesis pathways in *P. frutescens.*

## Materials and Methods

### Plant materials and sample preparation

The seeds of *P. frutescens* (L.) Britt accessions were purchased from the medicine market in Anguo City, Hebei Province, China, and stored at 4 °C in our lab. In the middle of March 2020, seeds were sown in a phytotron using plant nutritive soil (Miracle-Gro nutritive soil, Scotts Miracle-Gro, Marysville, OH USA). The plants were grown in a phytotron under controlled conditions (day/night, 10/14 h, 30/25 °C, 60% RH) in the greenhouse. When the height of the plant was approximately 50 cm, the young leaves (nodes 1 and 2), stems, and roots were harvested individually in the middle of June 2020 at three replications. This plant materials were *P. frutescens* (L.) from the College of pharmacy, Nanjing University of Chinese Medicine, indentified by the corresponding author of this article (Professor Qinan Wu). They were collected for RNA-Seq, GC–MS, and LC–MS analyses.

### Sample preparation

The volatile compounds were extracted from leaves, stems, and roots for 1 h using a modified simultaneous distillation extraction (SDE, modified Likens-Nickerson apparatus, Luxin, Shanghai, China). According to the preliminary experiments, 30 g of fresh tissue was accurately weighed and then mixed with deionized water in a 150 mL round-bottom flask. Then, it was attached to a modified Likens-Nickerson apparatus. The other round-bottom flask containing 50 mL of n-hexane was also connected to the apparatus. The round-bottom flasks were heated on both sides simultaneously. Water was passed through the condenser (DLSB-5L/25 cryogenic coolant circulating pump, Keer, Nanjing, China) containing 95% ethyl alcohol. The process was carried out for 1 h after the solvent in both flasks started to boil, and finally, the extract was collected in a brown bottle. Na_2_SO_4_ was added to the extract for dehydration. The supernatant was centrifuged and stored at -80 °C. For volatile oils in PGTs, we used the micropipette method. A capillary tube of 10 µL was drawn to form the tip of a tapered pipette using a needle puller (WD-1 microelectrode drawing instrument; Chendu, China). It was used to absorb volatile oil from PGTs on the abaxial surface of young leaves (node 1) under a stereomicroscope. Then, the tip of the capillary tube was broken into a liquid-phase vial containing 50 µL n-hexane. According to the preliminary experiments, the number of collected PGTs was approximately 240 and stored at -80 °C.

The caffeic acid standard was purchased from Desite (Industries Ltd., Chengdu, China). The rosmarinic acid standards were purchased from SenBeiJia Biological Technology Co., Ltd. (Nanjing, China). The perillaketone standard was purchased from TLC (TLC Pharmaceutical Standards Ltd., Newmarket, Ontario, Canada). The isoegomaketone standard was purchased from CFWLABS (CFW Laboratories, Inc., Newark, DE, USA).

### GC–MS conditions

An Agilent 7893A gas chromatograph coupled with an Agilent 7000C mass spectrometer (Agilent Technologies, Santa Clara, CA, USA) in electron impact mode was used for the qualitative and semiquantitative determination of the volatile compounds. The ionization voltage, injector temperature, and ion source temperature were 70 eV, 260 °C, and 250 °C, respectively. Mass spectra were scanned from 30 to 500 amu. Agilent 19091S-433 HP-5 ms (30 m × 250 μm × 0.25 μm; Agilent Technologies, Santa Clara, CA, USA) was used to separate the volatiles. The column temperature was initially 50 °C (held for 3 min), then raised to 100 °C at a rate of 10 °C/min (held for 3 min), and finally raised to 200 °C at 5 °C/min (held for 3 min). Helium was used as the carrier gas at a flow rate of 1.5 mL/min with an injection volume of 1 μL using a 20:1 split ratio. The compounds were analyzed qualitatively by comparing the retention times, the retention indices, and the mass spectra of the samples with those of standards, where available, and using the NIST 14.0 (NIST, Gaithersburg, Md., USA) libraries. The relative contents of the compounds were determined by the area normalization method calculated according to the following formula:

$$C{\text{i}} = \frac{Ai}{{\sum {Ai} }} \times 100\%$$

(Ci: relative contents of compound i; Ai: peak area of compound i).

### UPLC-ESI-Q-TOF–MS/MS conditions

According to the preliminary experiments, we weighed the leaves, stems, and roots (5.0 g) (accurate to 0.01 g). A total of 20 mL 80% methanol was added, and then tissues were extracted by ultrasonic extraction equipment (KH-300SP ultrasonic extraction equipment, Kunshanhechuang, China) three times for 30 min each. The extracts were diluted 10 times. The solutions were centrifuged at 13,000 rpm for 10 min and passed through a 0.22 μm filter membrane. The collection of PGT secretions was the same as GC–MS, which collected about 500 PGTs in 80% methanol based on our preliminary experiments [41–43].

The metabolite profiles were analyzed on a triple TOF 5600^+^ system (AB SCIEX, Foster City, CA, USA) with the ESI source coupled to a UPLC system (Shimadzu 30A UHPLC system, Shimadzu, Japan). For UPLC analysis, a 4 μL sample was injected into an analytical reverse-phase column (Agilent Extend-C18, 100 mm × 2.1 mm, 1.8 μm). The separation was performed with 0.1% formic acid in water and acetonitrile. The total running time was 34 min at a flow rate of 0.3 mL/min. The column compartment was maintained at 40 °C. The mobile phase used a gradient elution of 5%–70% acetonitrile (0–10 min), 70%–95% acetonitrile (10–28 min), 95% acetonitrile (28–30 min), and 4 min to return to the initial conditions of 5% acetonitrile.

For TOF analysis, the mass spectrometer was operated in the positive and negative ESI mode with a duo-spray source, and the mass scan range was set at m/z 50–2000 for both TOF–MS and TOF–MS/MS scans. The following parameters were used: ion spray voltage, 5500/-4500 V; turbo spray temperature, 550 °C; curtain gas, 35 psi; nebulizer gas, 55 °C; collision pressure, 40 V; collision energy spread, 20/-20 V; declustering potential, 60/-60 V; ion release delay, 66.63; and iron release width, 24.92. Analyst TF software (version 1.6, AB SCIEX, Foster City, CA, USA) combined with information-dependent acquisition packing was used to acquire MS/MS data. The spectra peaks were identified via the database from related literature of *Perilla* searched in CNKI, PUBMED, and SciFinder. Simultaneously, the mol files of the compounds were downloaded using Chemical Book, SciFinder, and PubChem and compared with Peakview software (version 1.2, AB SCIEX, Foster City, CA, USA).

### Scanning electron microscope (SEM) and stereomicroscope analysis

The morphology and distribution of the GTs were evaluated using a stereomicroscope (SetREO Discovery. V20, Zeiss, Jena, Germany) and SEM (SU3500, Hitachi, Tokyo, Japan). The tissues were fixed with a slide and observed using a stereomicroscope. For the SEM, leaves were fixed in glutaraldehyde (2.5% (v/v) in 0.1 M phosphate buffer (pH 7.3) for 12 h at 4 °C. They were then washed with a phosphate-buffered saline solution (Thermo Fisher, Shanghai, China) three times for 15 min each and dehydrated with an acetone dilution series (30, 50, 70, 90, and 100%). Acetone was replaced with tert-butanol overnight. The material was dried overnight in a freeze dryer (FreeZone 2.5 Liter Benchtop Freeze Dry System, Labconco, Kansas, USA). The specimens were then gold-coated (EM ACE200, Leica Microsystems, Leica Microsystems, Wetzlar, Germany). The coated specimens were then viewed and photographed using an SEM.

### RNA extension and transcriptome analysis

Total RNA from the three biological leaf replicates was extracted for RNA-Seq. Total RNA from four biological replicates from each of the plant parts (leaves, stems, and roots) was extracted for qRT-PCR. RNA degradation and contamination were monitored using 1% agarose gels. RNA purity was checked using a Nano Photometer spectrophotometer (Implen, Westlake Village, CA, USA). RNA concentration was measured using the Qubit RNA Assay Kit in Qubit 2.0 Fluorometer (Life Technologies, Carlsbad, CA, USA). RNA integrity was assessed using the RNA Nano 6000 Assay Kit of the Agilent Bioanalyzer 2100 system (Agilent Technologies, Santa Clara, CA, USA). cDNA library preparation for sequencing, data assessment, quality control, and RNA-seq assessment are provided in Supplementary file 1.

The remaining clean reads were de novo assembled into transcripts using Trinity (version 2.0.6) with default settings. Transcripts with a minimum length of 200 bp were clustered to minimize redundancy. For each cluster (representing the transcriptional complexity for the same gene), the longest sequence was preserved and designated as unigenes. Unigenes were blasted against the NCBI Nr (NCBI non-redundant protein database), SwissProt, TrEMBL, CDD (Conserved Domain Database), Pfam, and KOG (eukaryotic orthologous groups) databases (E-value < 1e-5). According to the priority order of the best-aligned results of NR, SwissProt and TrEMBL determined the Unigene ORF and the CDS and corresponding amino acid sequences according to the codon table. Simultaneously, TransDecoder (version 3.0.1) was used to predict CDS sequences of the un-aligned Unigenes. Gene Ontology (GO) functional annotation information was obtained according to the transcript annotation results of SwissProt and TrEMBL. KEGG (Kyoto Encyclopedia of Genes and Genomes) automatic annotation server (KAAS, version 2.1) was used for KEGG annotation.

### Identification of differentially expressed genes (DEGs)

Transcripts per million is a measure used to calculate the proportion of a transcript in an RNA pool. It takes into account the sequence depth and the length of the gene as well as the influence of the sample on the read count. Salmon (version 0.8.2) was used to calculate the read count and transcripts per million of the unigenes. DEGs were calculated based on the read counts of each gene. For samples with biological repetition, DESeq (version 1.12.4) was used for the analysis. To obtain significant differential genes, the screening conditions were set as follows: q-value < 0.05, and difference multiple |FoldChange|> 2. Functional enrichment analysis of the DEGs is shown in Supplementary File 1.

### Validation of qRT-PCR

To validate the accuracy of the transcriptome, 15 candidate genes involved in the flavonoid, rosmarinic acid, terpenoid biosynthetic pathways, and trichome development were selected and performed using QuantStudio 3 (Thermo Fisher Scientific Inc., Waltham, MA, USA). The qPCR protocol was 95 °C for 30 s, 95 °C for 10 s, and 60 °C for 30 s for 40 cycles, along with melting curve analysis. The gene-specific primers used in this study are listed in Supplementary Table S8. β-actin (TRINITY_DN47478_c3_g1) was used as an internal reference [44]. The 2^−∆∆CT^ method was used to calculate the relative gene expression levels. For each gene, four biological replicates were used in qRT-PCR experiments.

### Statistical analysis

Significant differences in gene expression were calculated using the paired-samples t-test with a significance level of *p* < 0.05, using SPSS version 24 (SPSS Inc., Chicago, IL, USA). All qRT-PCR expression analyses were performed in four replicates. The data were expressed as the mean ± standard error of the mean. Heatmaps were constructed using TBtools software (version 1.064).

## Supplementary Information

**Additional file 1: Supplementary Fig. 1**. The morphology of leaves (abaxial, A and adaxial, B surfaces), stems (D), and roots (C) of *P. frutescens *(bar=1 mm).

**Additional file 2: Supplementary Fig. 2**. The morphology of glandular trichomes in *P. frutescens *(bar=0.05 mm); A, B, C, D for PGTs; E, F, G, H for CGTs; I, J, K, L for DGTs.

**Additional file 3**: **Supplementary Fig. 3**. The measurement of glandular trichomes in *P. frutescens *(bar=0.05 mm); A, B for PGTs; C, D for DGTs; E, F for DGTs.

**Additional file 4: Supplementary Fig. 4**. GC-MS peaks of the essential oil extracts for leaves (A), PGTs (B), stems (C), roots (D) and their overlap peaks.

**Additional file 5: Supplementary Fig. 5**. LC-MS peaks of the essential oil extracts for leaves, stems, roots, and PGTs with positive and negative ESI mode; A for leaves pos, B for leaves neg; C for stems pos, D for stems neg; E for roots pos, F for roots neg; G for PGTs pos, H for PGTs neg.

**Additional file 6**: **Supplementary Fig. 6**. LC-MS peaks of the standard mixture and mass spectra of the standard mixture and extracts; Caffeic acid standard peaks (A) and mass spectra (B), Caffeic acid extract mass spectra (C); Rosmarinic acid standard peaks (D) and mass spectra (E), Rosmarinic acid extract mass spectra (F); Perillaketone standard peaks (G) and mass spectra (H), Perillaketone extract mass spectra (I); Isoegomaketone standard peaks (J) and mass spectra (K), Isoegomaketone extract mass spectra (L).

**Additional file 7**: **Supplementary Fig. 7**. Species distribution of *P. frutescens *homologues against the Nr database.

**Additional file 8**: **Supplementary Fig. 8**. GO (A) and KEGG (B) analysis of *P. frutescens.*

**Additional file 9**: **Supplementary Fig. 9**. The DEGs barplot (A) and DEGs venn (B) of *P. frutescens.*

**Additional file 10**: **Supplementary Fig. 10**. GO classifications and KEGG pathway of S vs R (A, D), L vs S (B,E) and L vs R (C, F).

**Additional file 11**: **Supplementary Fig. 11**. Heatmap of trichome branching (A), trichome differentiation(B) and trichome morphogenesis (C).

**Additional file 12**: **Supplementary Fig. 12**. Classifications based on metabolism of terpenoids and polyketide.

**Additional file 13**: **Supplementary Fig. 13**. Metabolites and Other DEGs in phenylpropanoid biosynthesis (A, B).

**Additional file 14**: **Supplementary Fig. 14**. The expression of genes in GA and JA pathway. Unigenes levels data represent by TPM. Red and green represent high and low expression levels, respectively.

**Additional file 15: Supplementary Fig. 15**. Possible reaction steps controlled by genes in hypothetical biosynthetic pathways of chemotypes of *P. frutescens *(Yuba, Honda, Koezuka & Tabata, 1995)*.*

**Additional file 16: Supplementary Fig. 16**. Stem transverse section of *P. frutescens* under bright field.

**Additional file 17: Table S1**. Metabolite profile in of *P. frutescens* by UPLC-ESI-Q-TOF-MS/MS analysis. **Table S2**. All sample raw data information. **Table S3**. All sample clean data information. **Table S4**. Assembly result ofclean reads. **Table S5**. Annotation ratio of 7 datebase. **Table S6**. GO anniotation of DEGs in three comparation. **Table S7**. Unigenes involved in the terpenoid biosynthesis pathway in *P. frutescens*. **Table S8**. Specific primers for qPCR. **Table S9**. Details for qRT-PCR and RNA-Seq. **Table S10**. The chemotypes of *P. frutescens.*

**Additional file 18: Methods**.

## Data Availability

Raw RNA-seq data of leaves, stems, and roots are available from the NCBI Sequence Read Archive (SRA; accession number: PRJNA690131, https://www.ncbi.nlm.nih.gov/bioproject/PRJNA690131).

## Abbreviations

GTs: Glandular trichomes
PGTs: Peltate glandular trichomes
CGTs: Capitate glandular trichomes
DGTs: Digitiform glandular trichomes
NGTs: Non-glandular trichomes
SDE: Simultaneous distillation extraction
CDD: Conserved Domain Database
GO: Gene Ontology
DEGs: Differentially expressed genes
