# Supplementary material for: Multi-omics analysis of the bioactive constituents biosynthesis of glandular trichome in Perilla frutescens
Source: BMC Plant Biol. 2021 Jun 18;21:277. doi: 10.1186/s12870-021-03069-4 (PMC8214284; doi:10.1186/s12870-021-03069-4)
Supplement: Supplementary file 1 — Additional file 1: Supplementary Fig. 1. The morphology of leaves (abaxial, A and adaxial, B surfaces), stems (D), and roots (C) of P. frutescens (bar=1 mm). [file 12870_2021_3069_MOESM1_ESM.pdf]

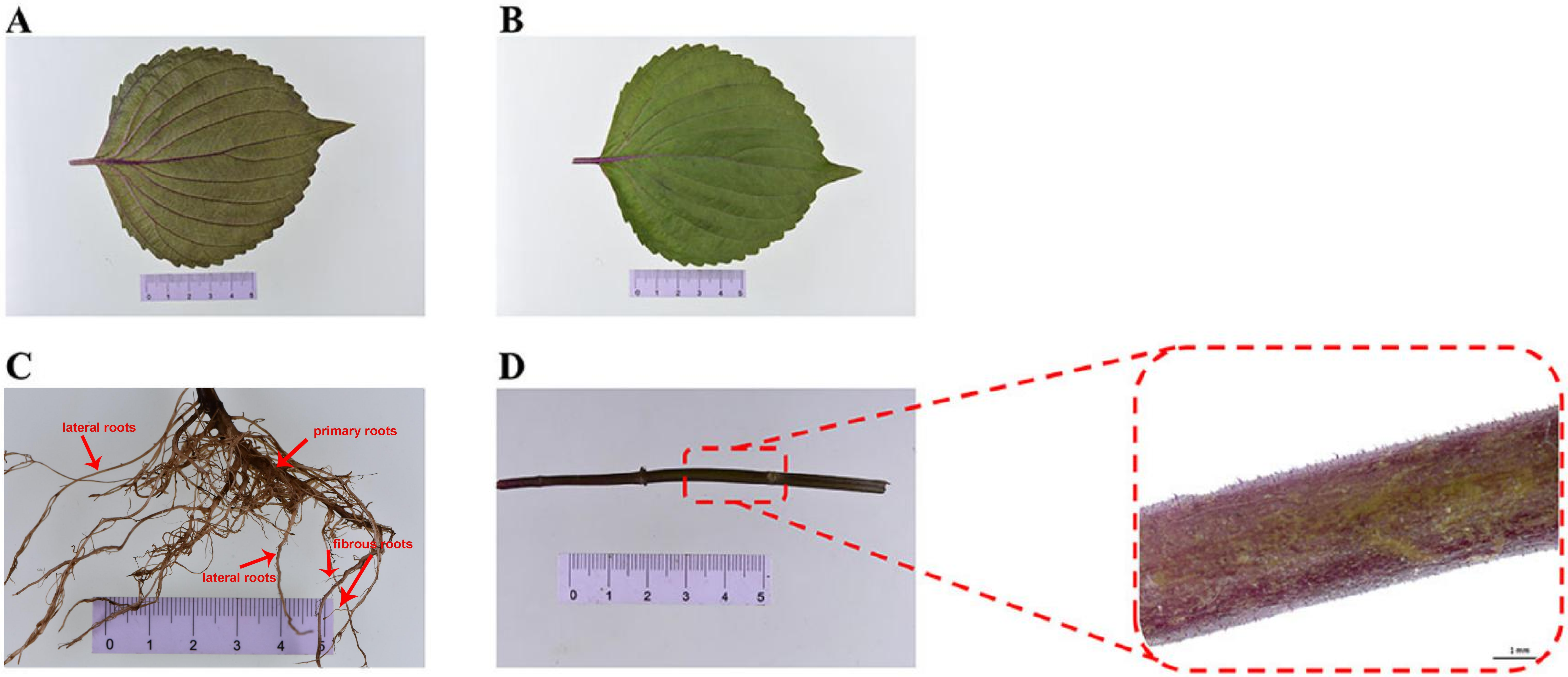

Supplementary Fig.1. The morphology of leaves (abaxial, A and adaxial, B surfaces), stems (D), and roots (C) of *P. frutescens* (bar=1 mm);
