# Supplementary material for: Multi-omics analysis of the bioactive constituents biosynthesis of glandular trichome in Perilla frutescens
Source: BMC Plant Biol. 2021 Jun 18;21:277. doi: 10.1186/s12870-021-03069-4 (PMC8214284; doi:10.1186/s12870-021-03069-4)
Supplement: Supplementary file 2 — Additional file 2: Supplementary Fig. 2. The morphology of glandular trichomes in P. frutescens (bar=0.05 mm); A, B, C, D for PGTs; E, F, G, H for CGTs; I, J, K, L for DGTs. [file 12870_2021_3069_MOESM2_ESM.pdf]

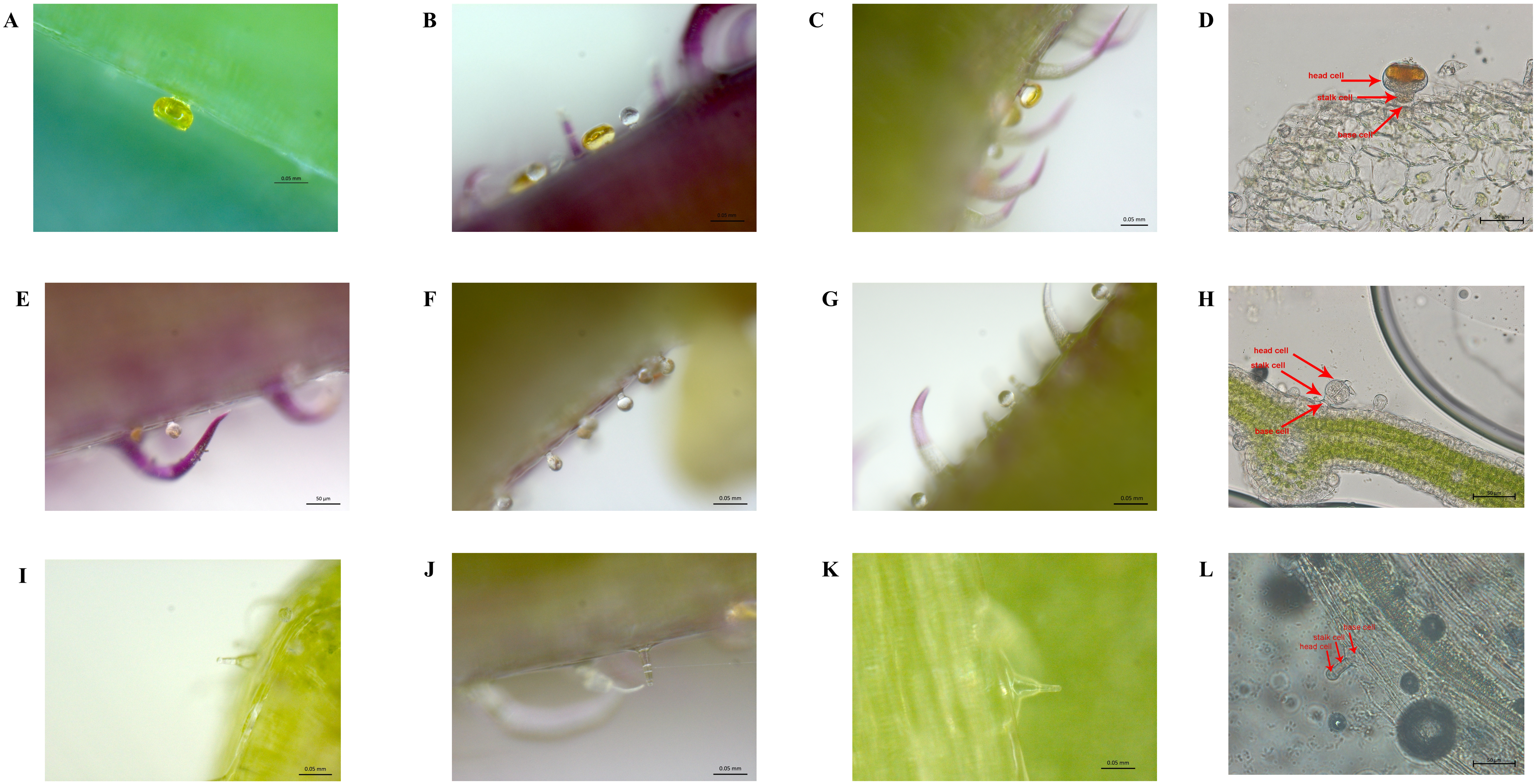

Supplementary Fig.2. The morphology of glandular trichomes in *P. frutescens* (bar=0.05 mm); A, B, C, D for PGTs; E, F, G, H for CGTs; I, J, K, L for DGTs.
