# Supplementary material for: Multi-omics analysis of the bioactive constituents biosynthesis of glandular trichome in Perilla frutescens
Source: BMC Plant Biol. 2021 Jun 18;21:277. doi: 10.1186/s12870-021-03069-4 (PMC8214284; doi:10.1186/s12870-021-03069-4)
Supplement: Supplementary file 3 — Additional file 3: Supplementary Fig. 3. The measurement of glandular trichomes in P. frutescens (bar=0.05 mm); A, B for PGTs; C, D for DGTs; E, F for DGTs. [file 12870_2021_3069_MOESM3_ESM.pdf]

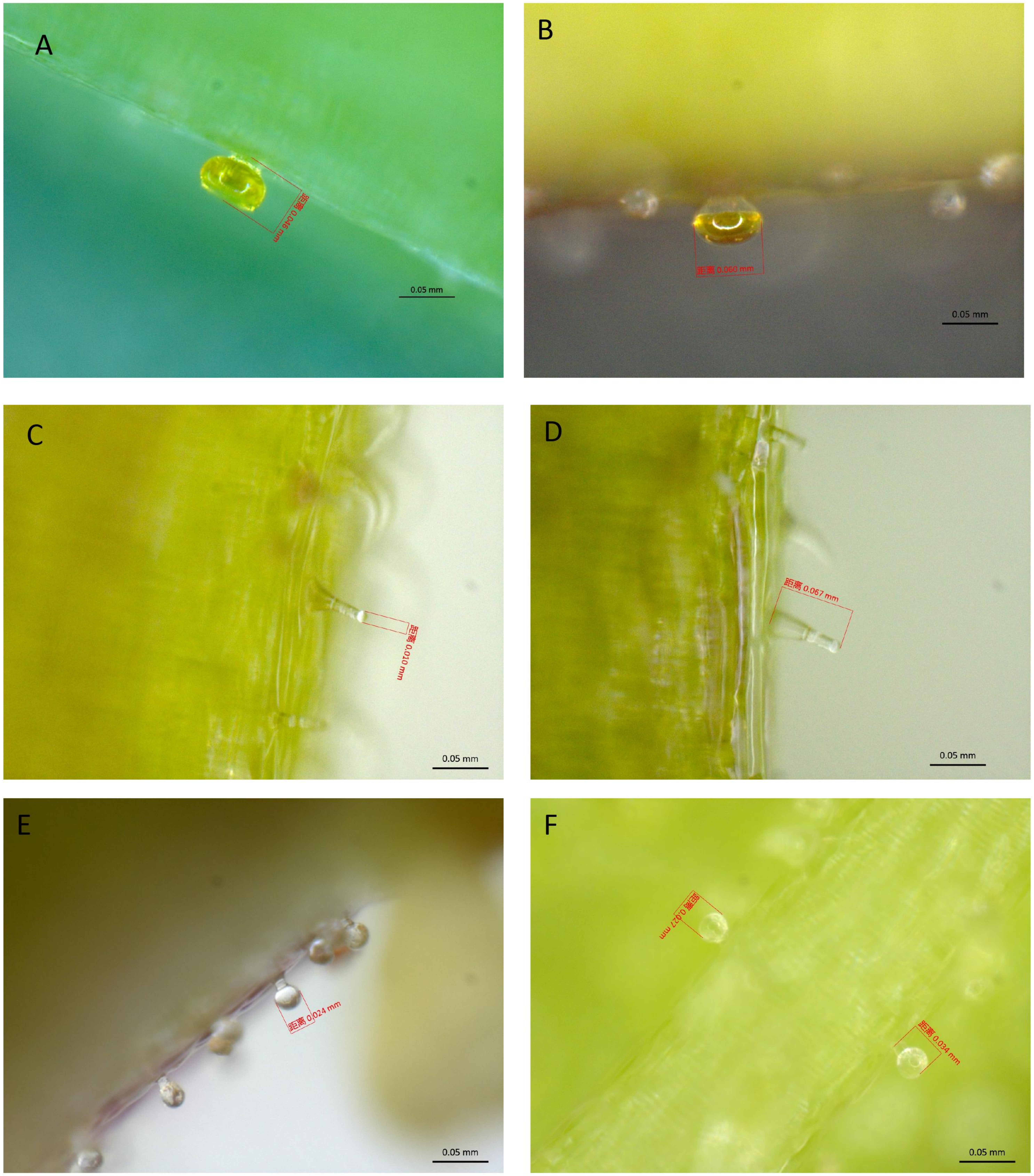

Supplementary Fig.3. The measurement of glandular trichomes in *P. frutescens* (bar=0.05 mm); A, B for PGTs; C, D for DGTs; E, F for DGTs.
