# Supplementary material for: Multi-omics analysis of the bioactive constituents biosynthesis of glandular trichome in Perilla frutescens
Source: BMC Plant Biol. 2021 Jun 18;21:277. doi: 10.1186/s12870-021-03069-4 (PMC8214284; doi:10.1186/s12870-021-03069-4)
Supplement: Supplementary file 4 — Additional file 4: Supplementary Fig. 4. GC-MS peaks of the essential oil extracts for leaves (A), PGTs (B), stems (C), roots (D) and their overlap peaks. [file 12870_2021_3069_MOESM4_ESM.pdf]

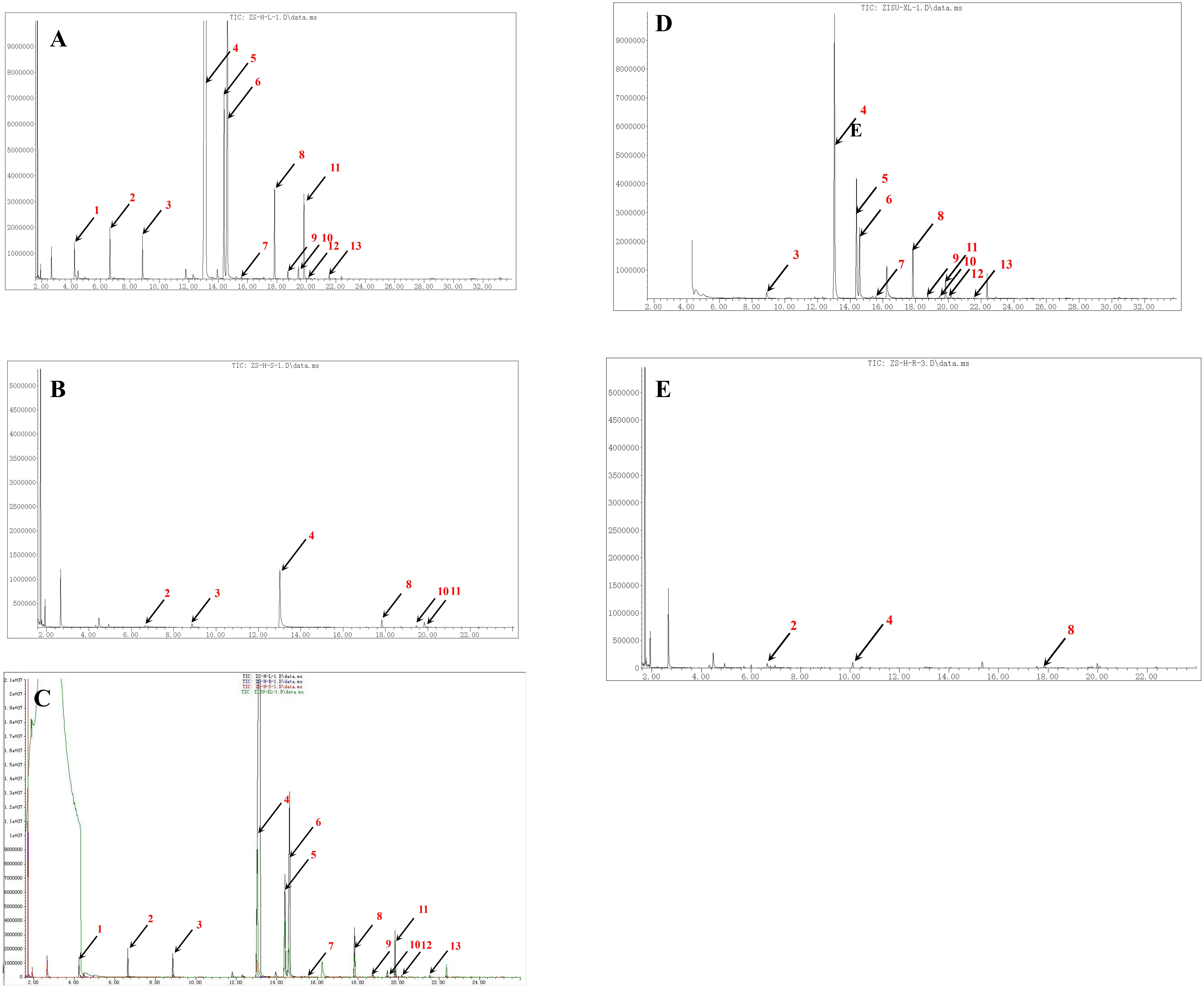

Supplementary Fig.4. GC-MS peaks of the essential oil extracts for leaves (A), PGTs (B), stems (C), roots (D) and their overlap peaks.
