# Supplementary material for: Multi-omics analysis of the bioactive constituents biosynthesis of glandular trichome in Perilla frutescens
Source: BMC Plant Biol. 2021 Jun 18;21:277. doi: 10.1186/s12870-021-03069-4 (PMC8214284; doi:10.1186/s12870-021-03069-4)
Supplement: Supplementary file 5 — Additional file 5: Supplementary Fig. 5. LC-MS peaks of the essential oil extracts for leaves, stems, roots, and PGTs with positive and negative ESI mode; A for leaves pos, B for leaves neg; C for stems pos, D for stems neg; E for roots pos, F for roots neg; G for PGTs pos, H for PGTs neg. [file 12870_2021_3069_MOESM5_ESM.pdf]

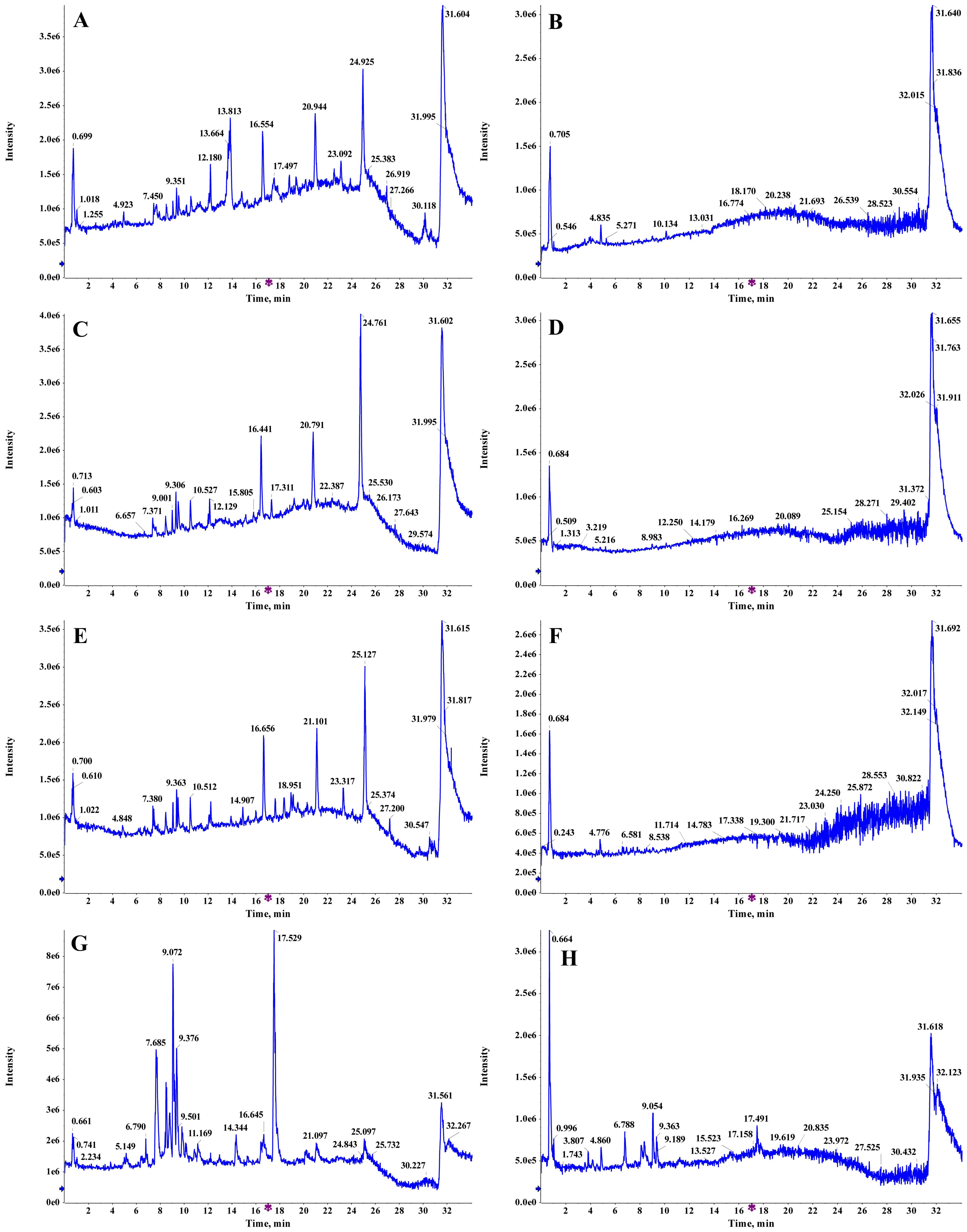


**Supplementary Fig.5. LC-MS peaks of the essential oil extracts for leaves, stems, roots, and PGTs with positive and negative ESI mode; A for leaves pos, B for leaves neg; C for stems pos, D for stems neg; E for roots pos, F for roots neg; G for PGTs pos, H for PGTs neg.**
