# Supplementary material for: Multi-omics analysis of the bioactive constituents biosynthesis of glandular trichome in Perilla frutescens
Source: BMC Plant Biol. 2021 Jun 18;21:277. doi: 10.1186/s12870-021-03069-4 (PMC8214284; doi:10.1186/s12870-021-03069-4)
Supplement: Supplementary file 6 — Additional file 6: Supplementary Fig. 6. LC-MS peaks of the standard mixture and mass spectra of the standard mixture and extracts; Caffeic acid standard peaks (A) and mass spectra (B), Caffeic acid extract mass spectra (C); Rosmarinic acid standard peaks (D) and mass spectra (E), Rosmarinic acid extract mass spectra (F); Perillaketone standard peaks (G) and mass spectra (H), Perillaketone extract mass spectra (I); Isoegomaketone standard peaks (J) and mass spectra (K), Isoegomaketone extract mass spectra (L). [file 12870_2021_3069_MOESM6_ESM.pdf]

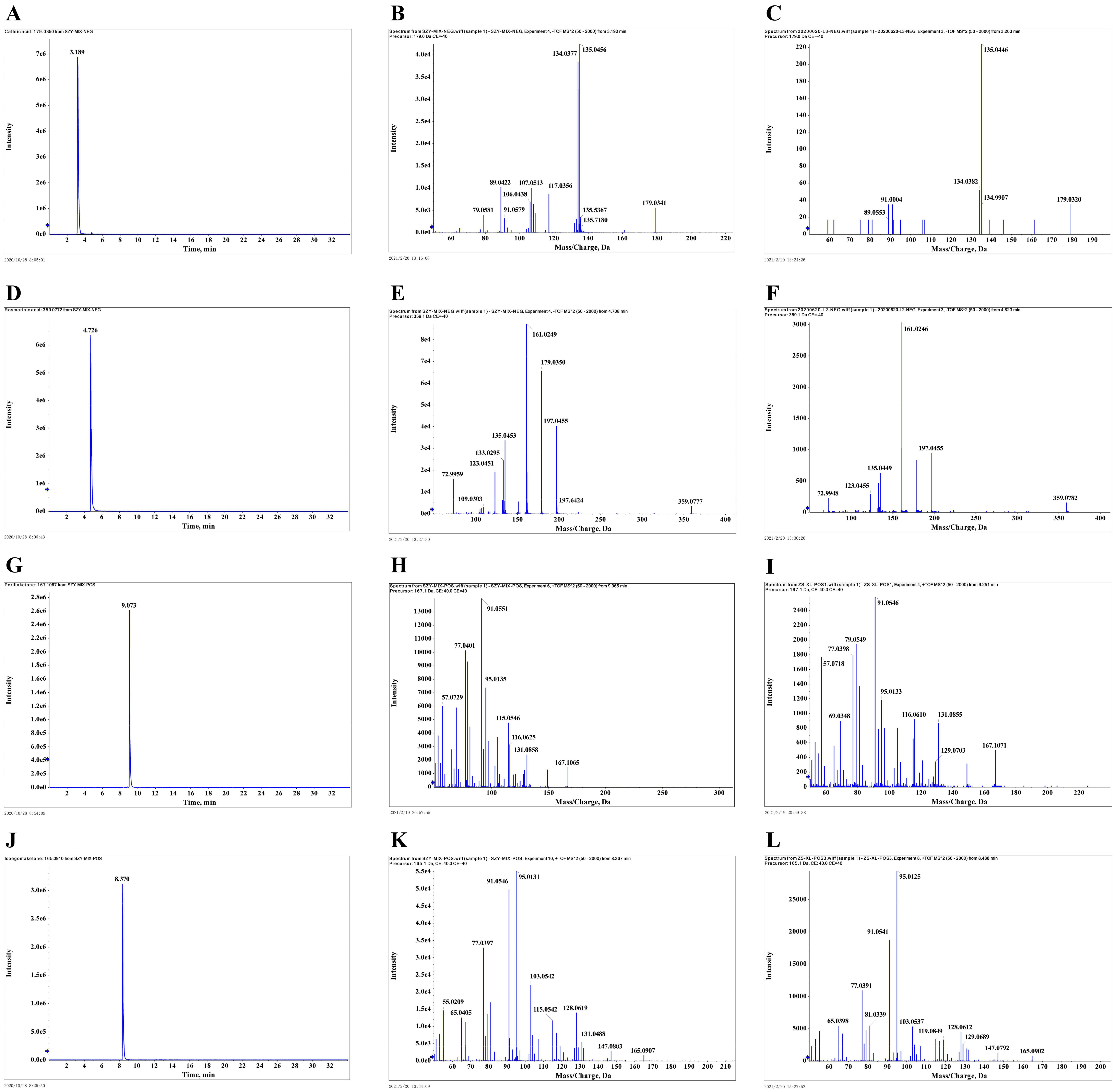

Supplementary Fig.6. LC-MS peaks of the standard mixture and mass spectra of the standard mixture and extracts; Caffeic acid standard peaks (A) and mass spectra (B), Caffeic acid extract mass spectra (C); Rosmarinic acid standard peaks (D) and mass spectra (E), Rosmarinic acid extract mass spectra (F); Perillaketone standard peaks (G) and mass spectra (H), Perillaketone extract mass spectra (I); Isoegomaketone standard peaks (J) and mass spectra (K), Isoegomaketone extract mass spectra (L).
