# Supplementary figures and images for: Multi-omics analysis of the bioactive constituents biosynthesis of glandular trichome in Perilla frutescens
Source: BMC Plant Biol. 2021 Jun 18;21:277. doi: 10.1186/s12870-021-03069-4 (PMC8214284; doi:10.1186/s12870-021-03069-4)

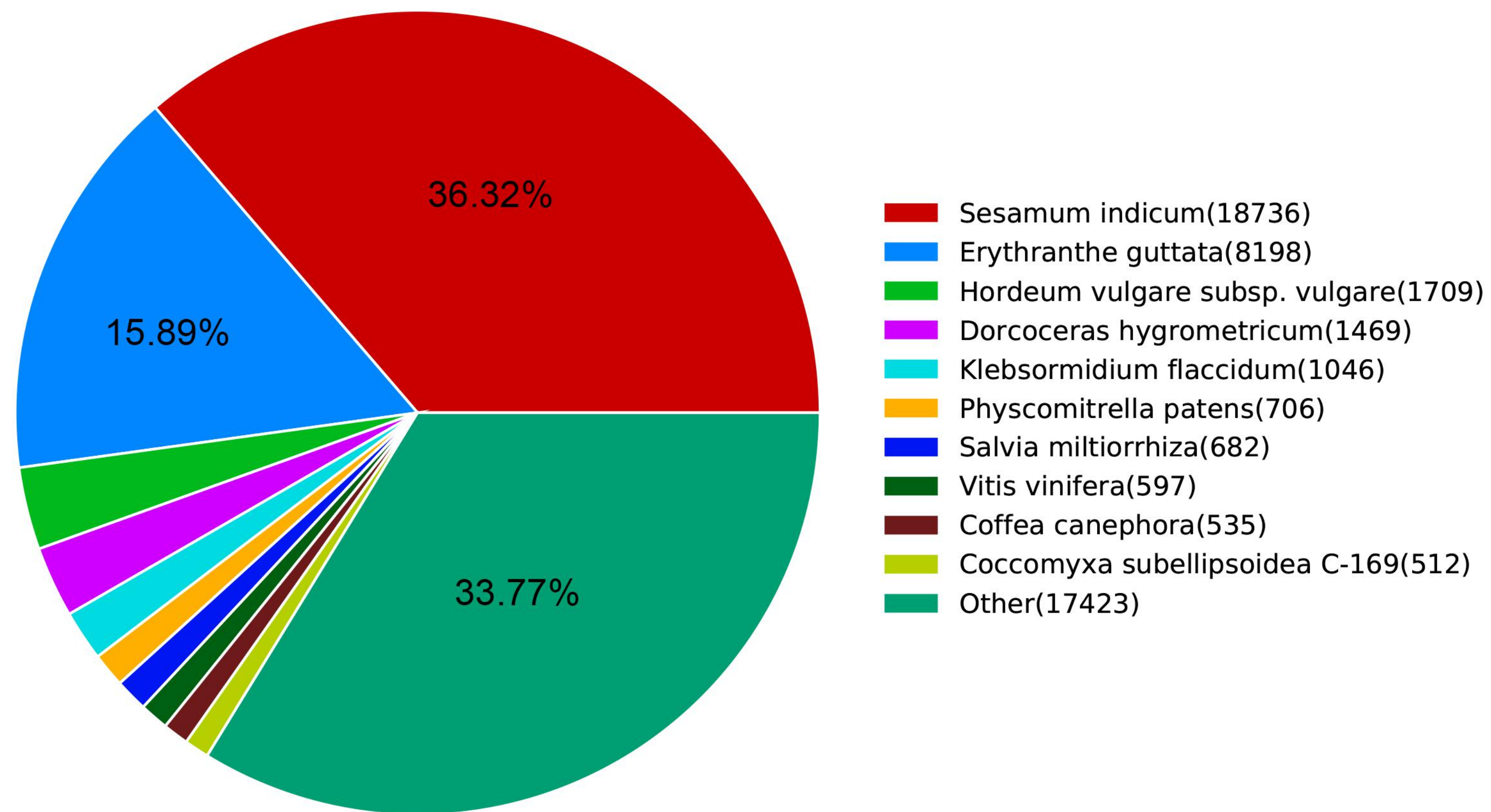

**Supplementary Fig.7. Species distribution of *P. frutescens* homologues against the Nr database.**

Supplement: Supplementary file 7 — Additional file 7: Supplementary Fig. 7. Species distribution of P. frutescens homologues against the Nr database. [file 12870_2021_3069_MOESM7_ESM.pdf]

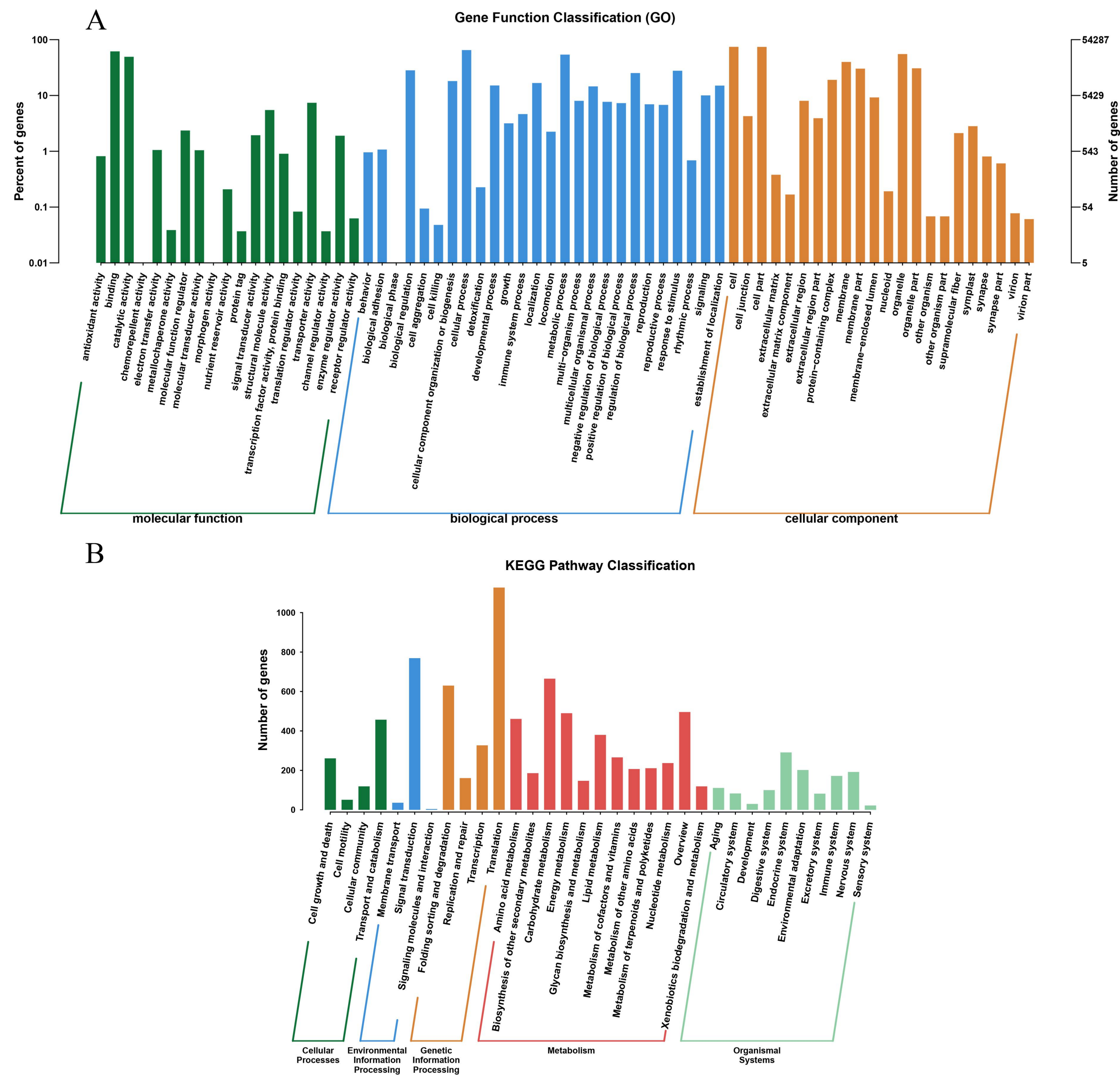

**Supplementary Fig.8. GO (A) and KEGG (B) analysis of *P. frutescens*.**

Supplement: Supplementary file 8 — Additional file 8: Supplementary Fig. 8. GO (A) and KEGG (B) analysis of P. frutescens. [file 12870_2021_3069_MOESM8_ESM.pdf]

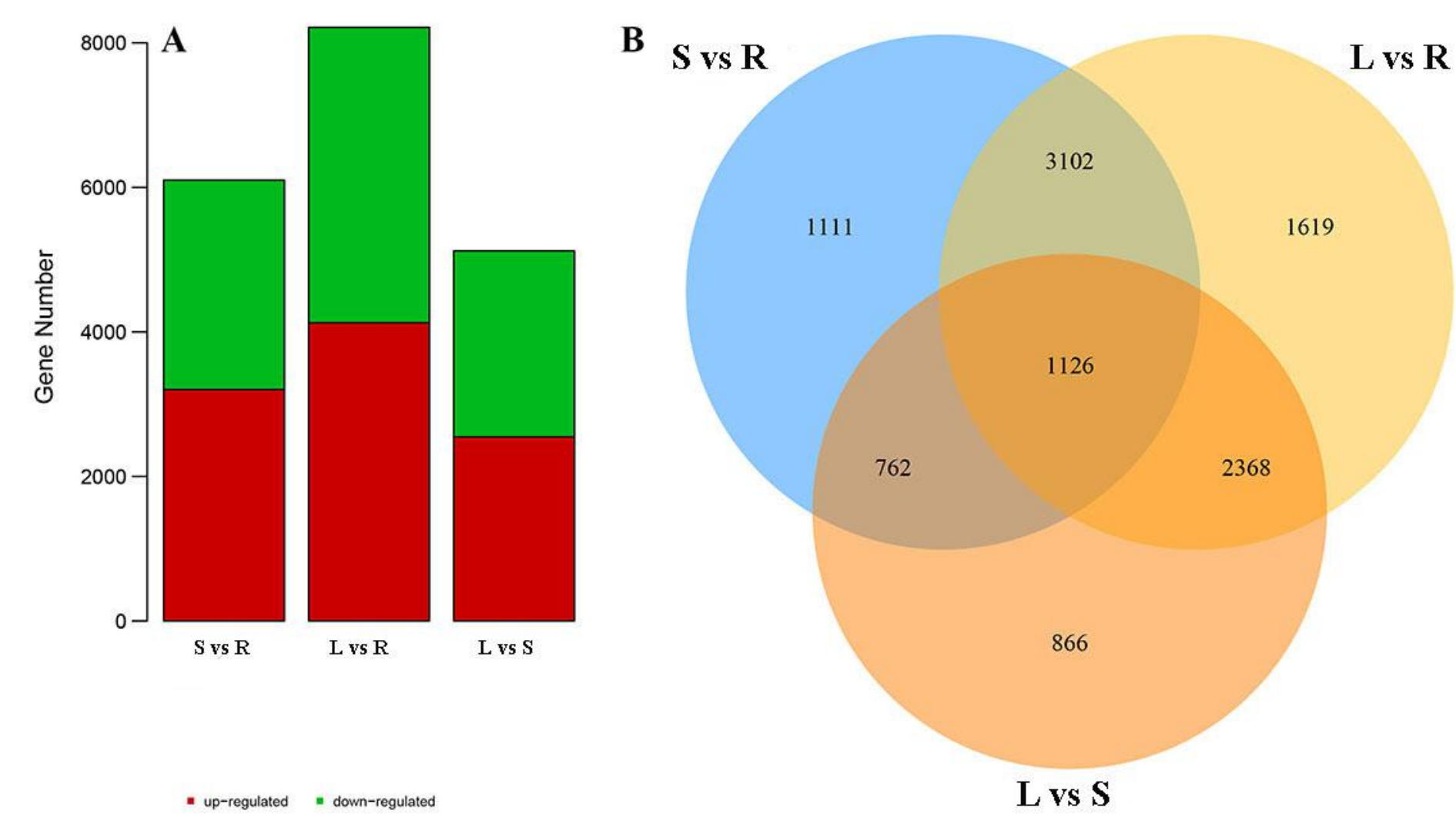

**Supplementary Fig.9. The DEGs barplot (A) and DEGs venn (B) of *P. frutescens*.**

Supplement: Supplementary file 9 — Additional file 9: Supplementary Fig. 9. The DEGs barplot (A) and DEGs venn (B) of P. frutescens. [file 12870_2021_3069_MOESM9_ESM.pdf]

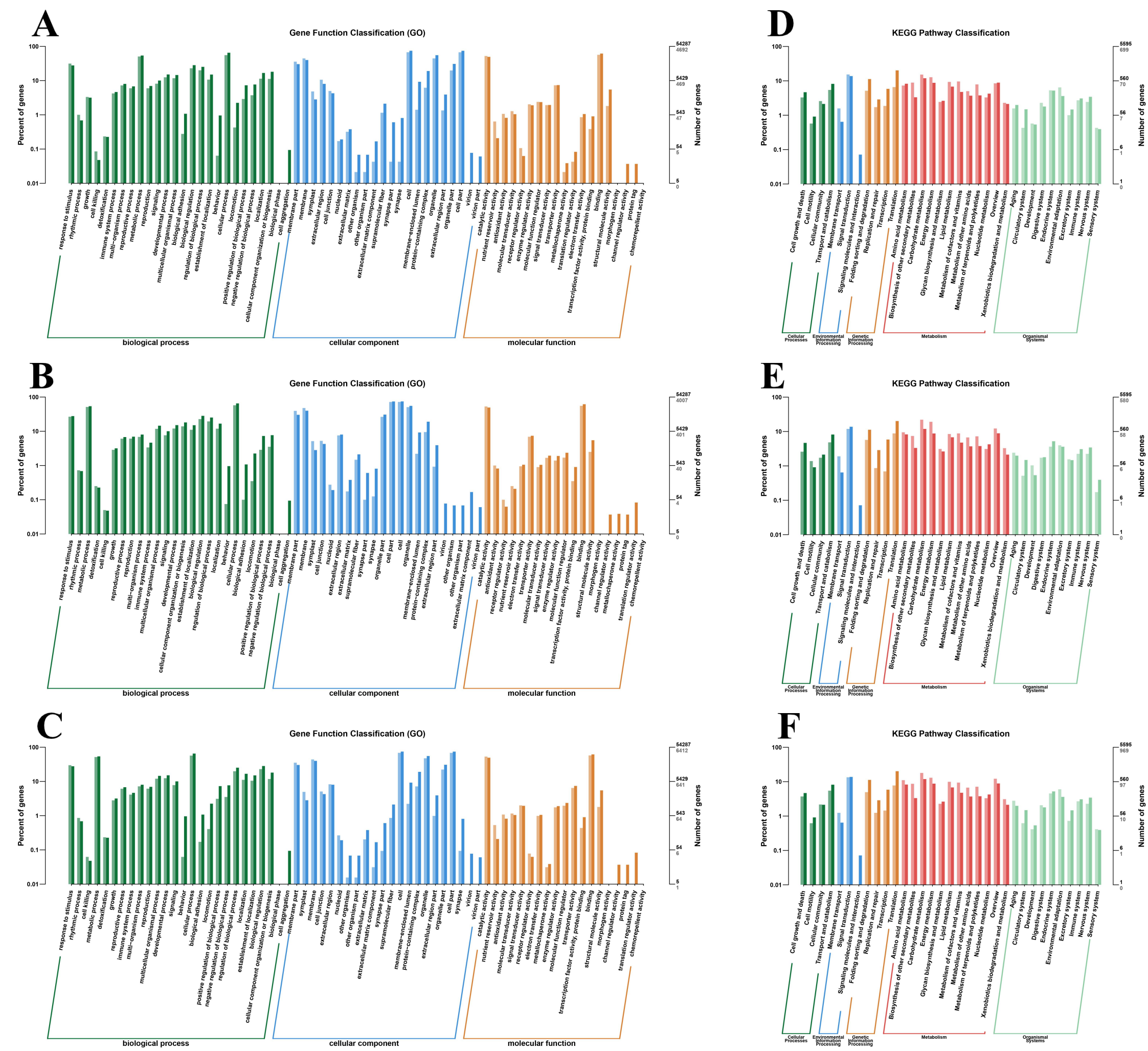

**Supplementary Fig.10. GO classifications and KEGG pathway of S vs R (A, D), L vs S (B,E) and L vs R (C, F).**

Supplement: Supplementary file 10 — Additional file 10: Supplementary Fig. 10. GO classifications and KEGG pathway of S vs R (A, D), L vs S (B,E) and L vs R (C, F). [file 12870_2021_3069_MOESM10_ESM.pdf]

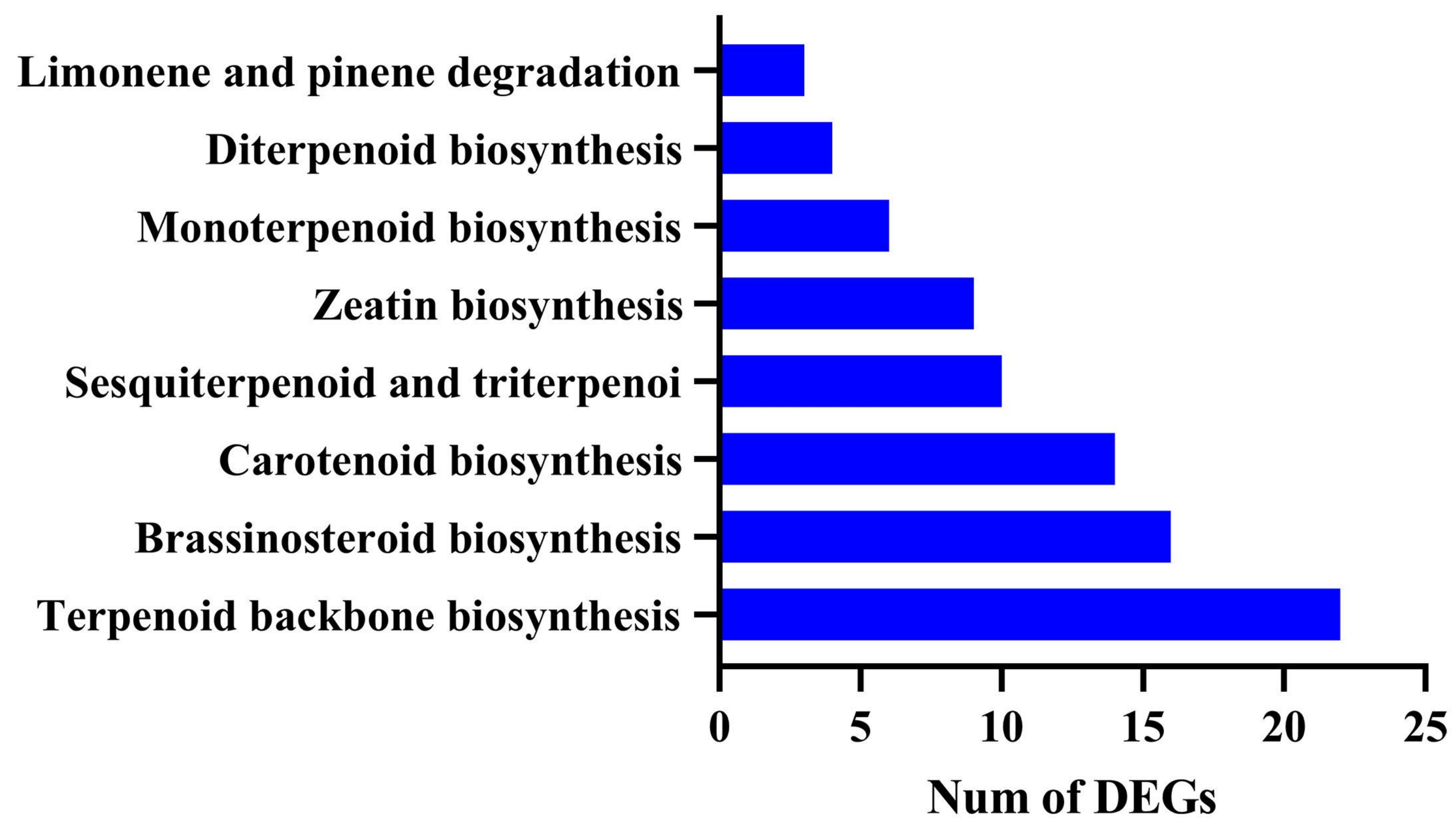

**Supplementary Fig.12. Classifications based on metabolism of terpenoids and polyketide.**

Supplement: Supplementary file 12 — Additional file 12: Supplementary Fig. 12. Classifications based on metabolism of terpenoids and polyketide. [file 12870_2021_3069_MOESM12_ESM.pdf]

A

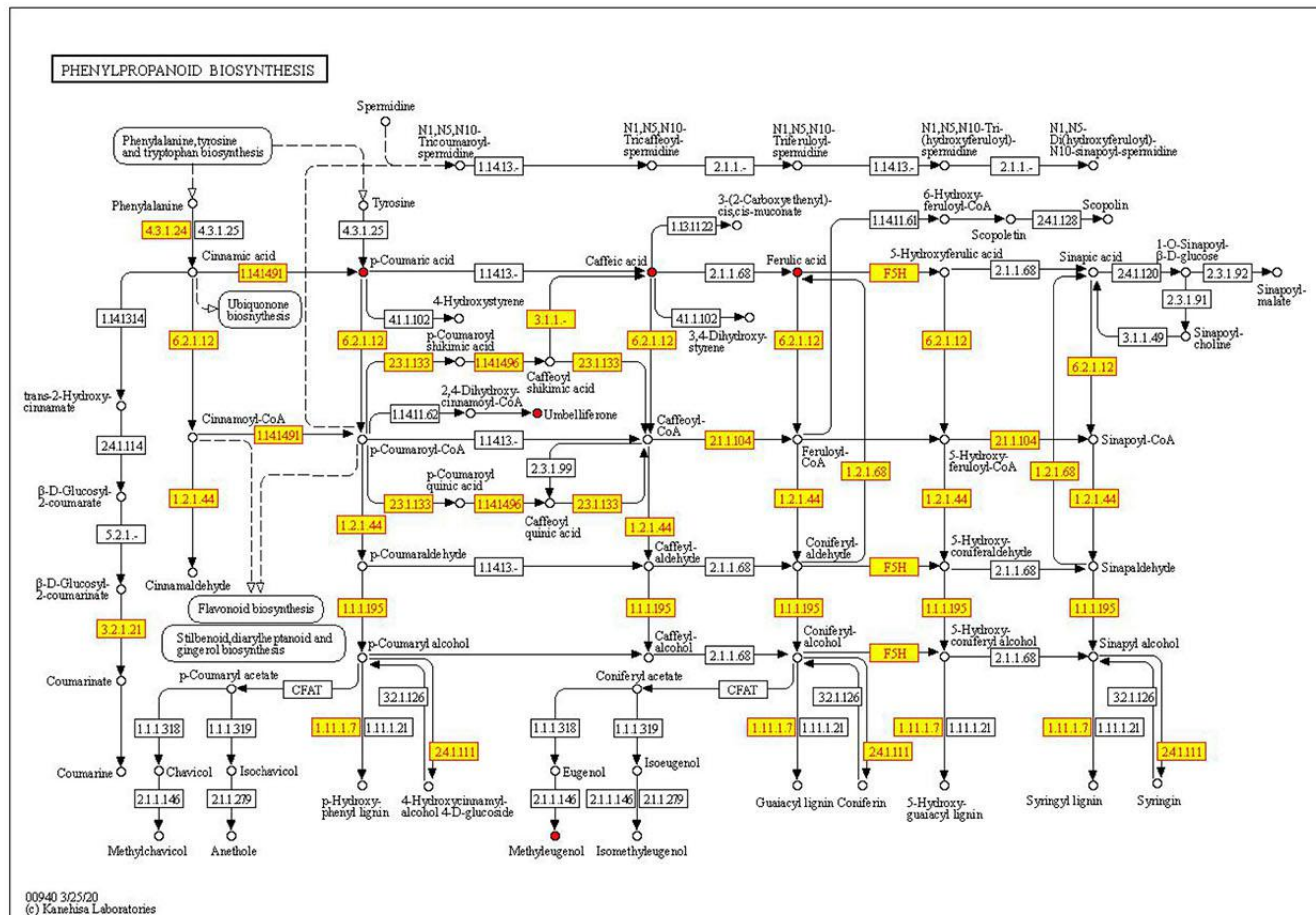

B

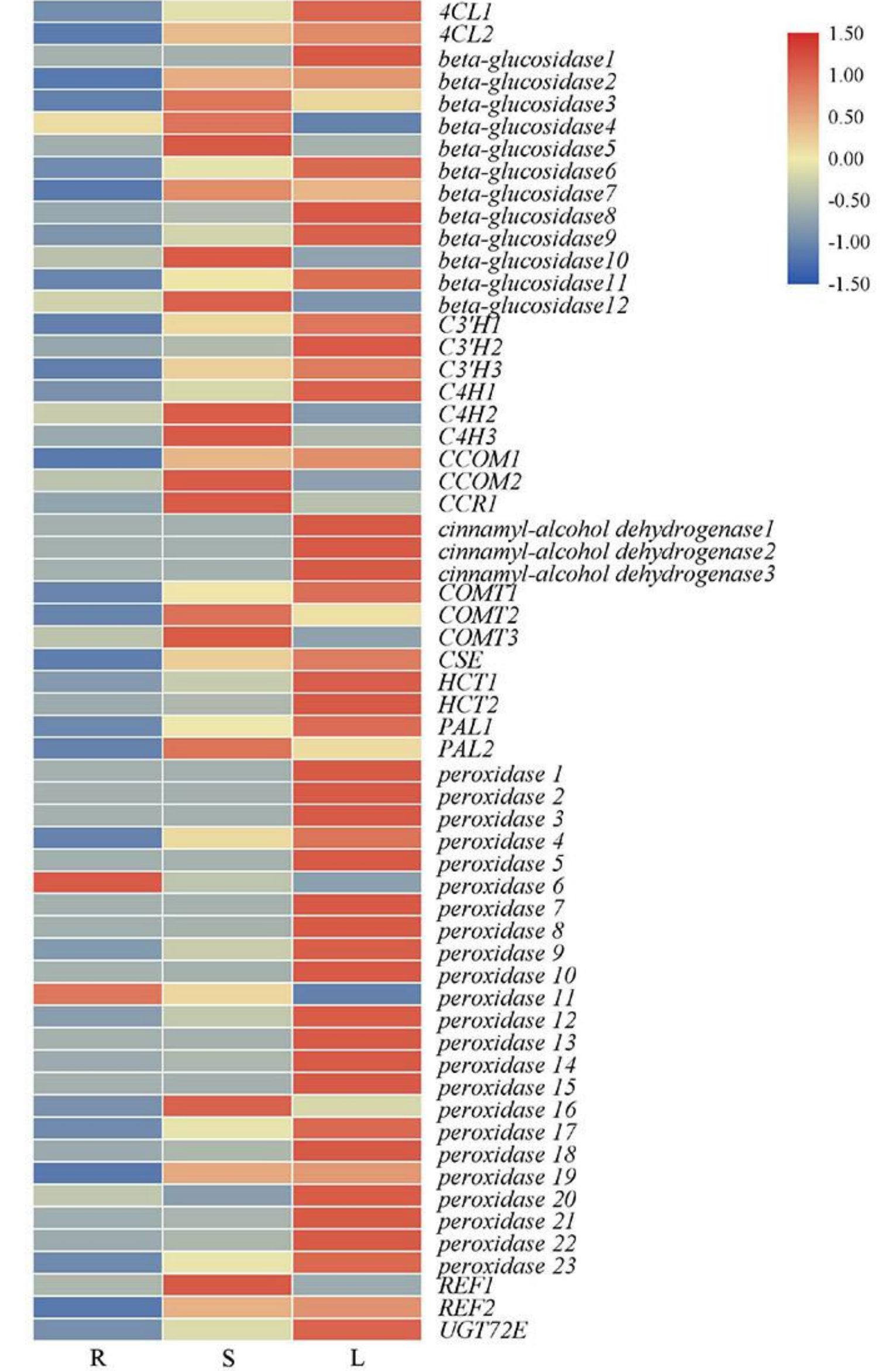

Supplementary Fig.13. Metabolites and Other DEGs in phenylpropanoid biosynthesis (A, B).

Supplement: Supplementary file 13 — Additional file 13: Supplementary Fig. 13. Metabolites and Other DEGs in phenylpropanoid biosynthesis (A, B). [file 12870_2021_3069_MOESM13_ESM.pdf]

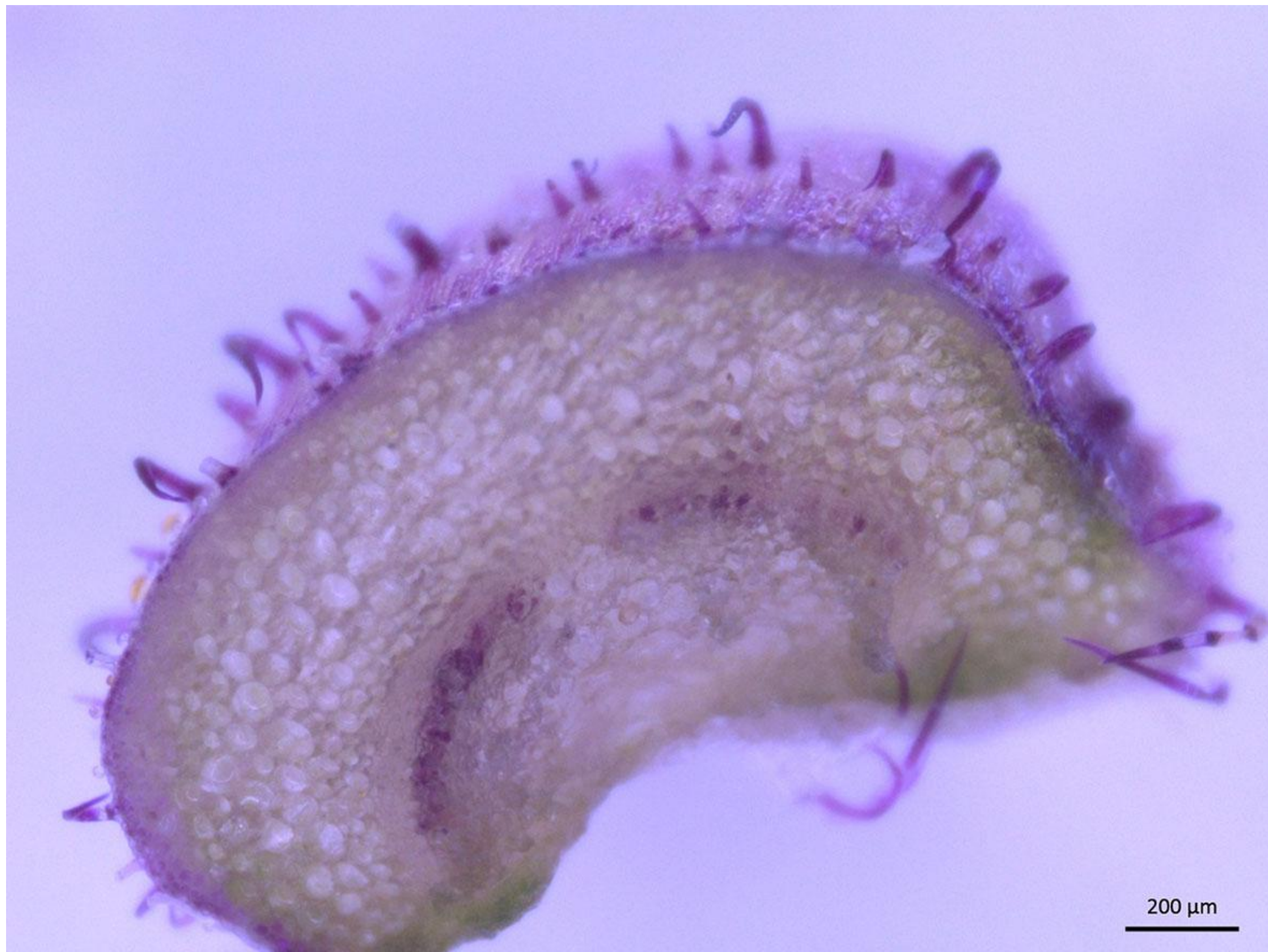

**Supplementary Fig.16. Stem transverse section of *P. frutescens* under bright field.**

Supplement: Supplementary file 16 — Additional file 16: Supplementary Fig. 16. Stem transverse section of P. frutescens under bright field. [file 12870_2021_3069_MOESM16_ESM.pdf]
