# Supplementary material for: Multi-omics analysis of the bioactive constituents biosynthesis of glandular trichome in Perilla frutescens
Source: BMC Plant Biol. 2021 Jun 18;21:277. doi: 10.1186/s12870-021-03069-4 (PMC8214284; doi:10.1186/s12870-021-03069-4)
Supplement: Supplementary file 11 — Additional file 11: Supplementary Fig. 11. Heatmap of trichome branching (A), trichome differentiation(B) and trichome morphogenesis (C). [file 12870_2021_3069_MOESM11_ESM.pdf]

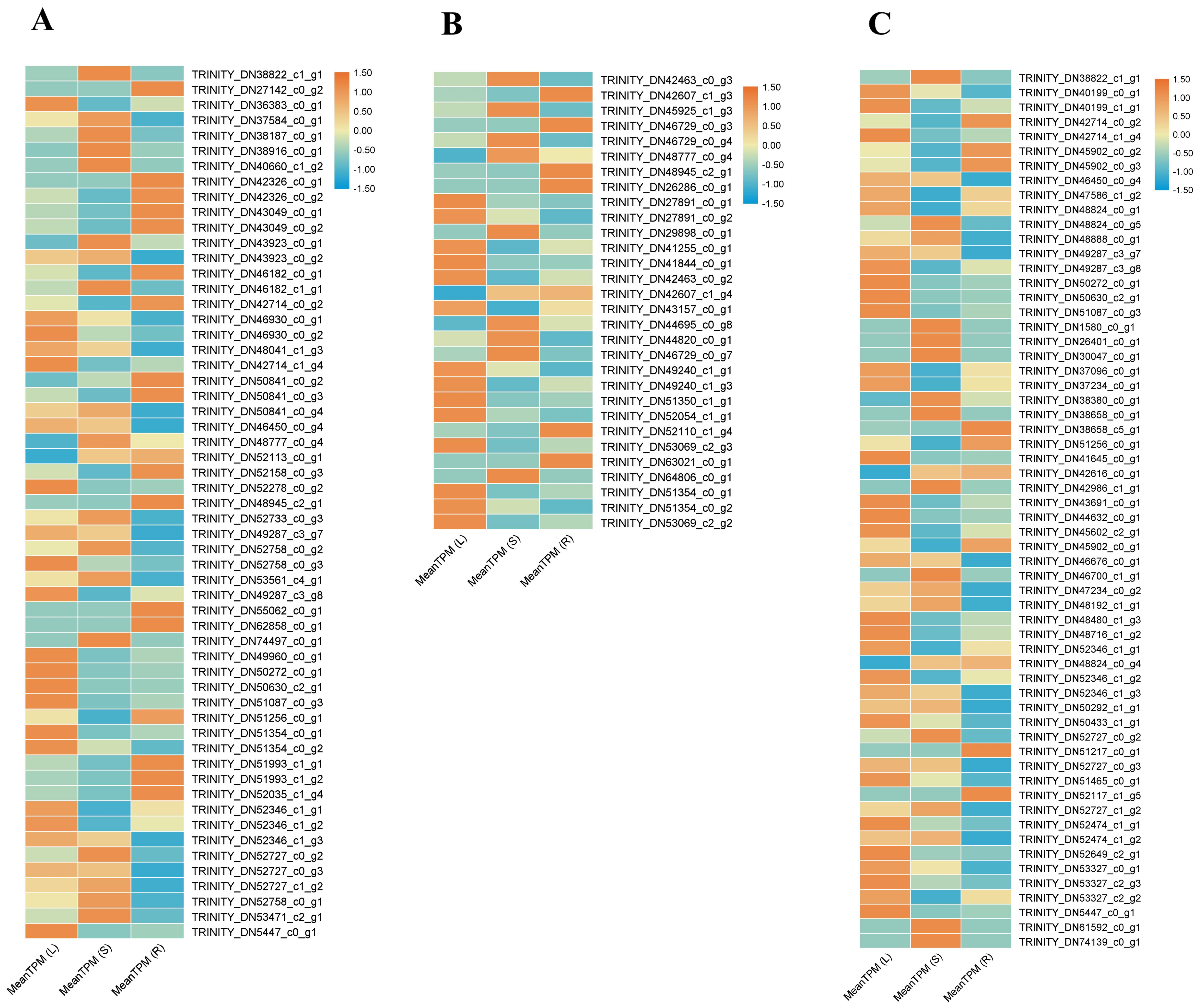

Supplementary Fig.11. Heatmap of trichome branching (A), trichome differentiation(B) and trichome morphogenesis (C).
