# Supplementary material for: Multi-omics analysis of the bioactive constituents biosynthesis of glandular trichome in Perilla frutescens
Source: BMC Plant Biol. 2021 Jun 18;21:277. doi: 10.1186/s12870-021-03069-4 (PMC8214284; doi:10.1186/s12870-021-03069-4)
Supplement: Supplementary file 14 — Additional file 14: Supplementary Fig. 14. The expression of genes in GA and JA pathway. Unigenes levels data represent by TPM. Red and green represent high and low expression levels, respectively. [file 12870_2021_3069_MOESM14_ESM.pdf]

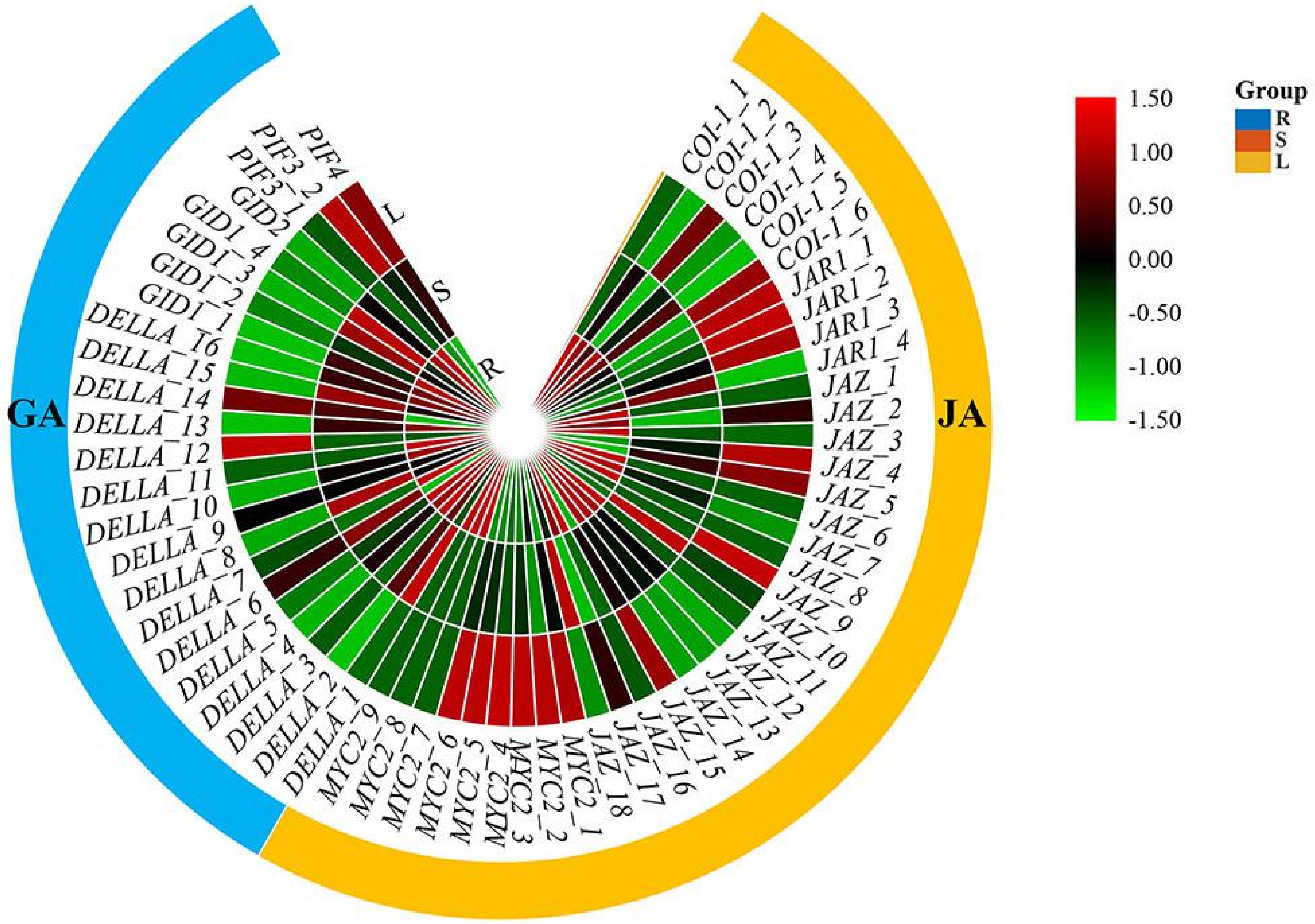

Supplementary Fig.14. The expression of genes in GA and JA pathway. Unigenes levels data represent by TPM. Red and green represent high and low expression levels, respectively.
