# Supplementary material for: Multi-omics analysis of the bioactive constituents biosynthesis of glandular trichome in Perilla frutescens
Source: BMC Plant Biol. 2021 Jun 18;21:277. doi: 10.1186/s12870-021-03069-4 (PMC8214284; doi:10.1186/s12870-021-03069-4)
Supplement: Supplementary file 15 — Additional file 15: Supplementary Fig. 15. Possible reaction steps controlled by genes in hypothetical biosynthetic pathways of chemotypes of P. frutescens (Yuba, Honda, Koezuka & Tabata, 1995). [file 12870_2021_3069_MOESM15_ESM.pdf]

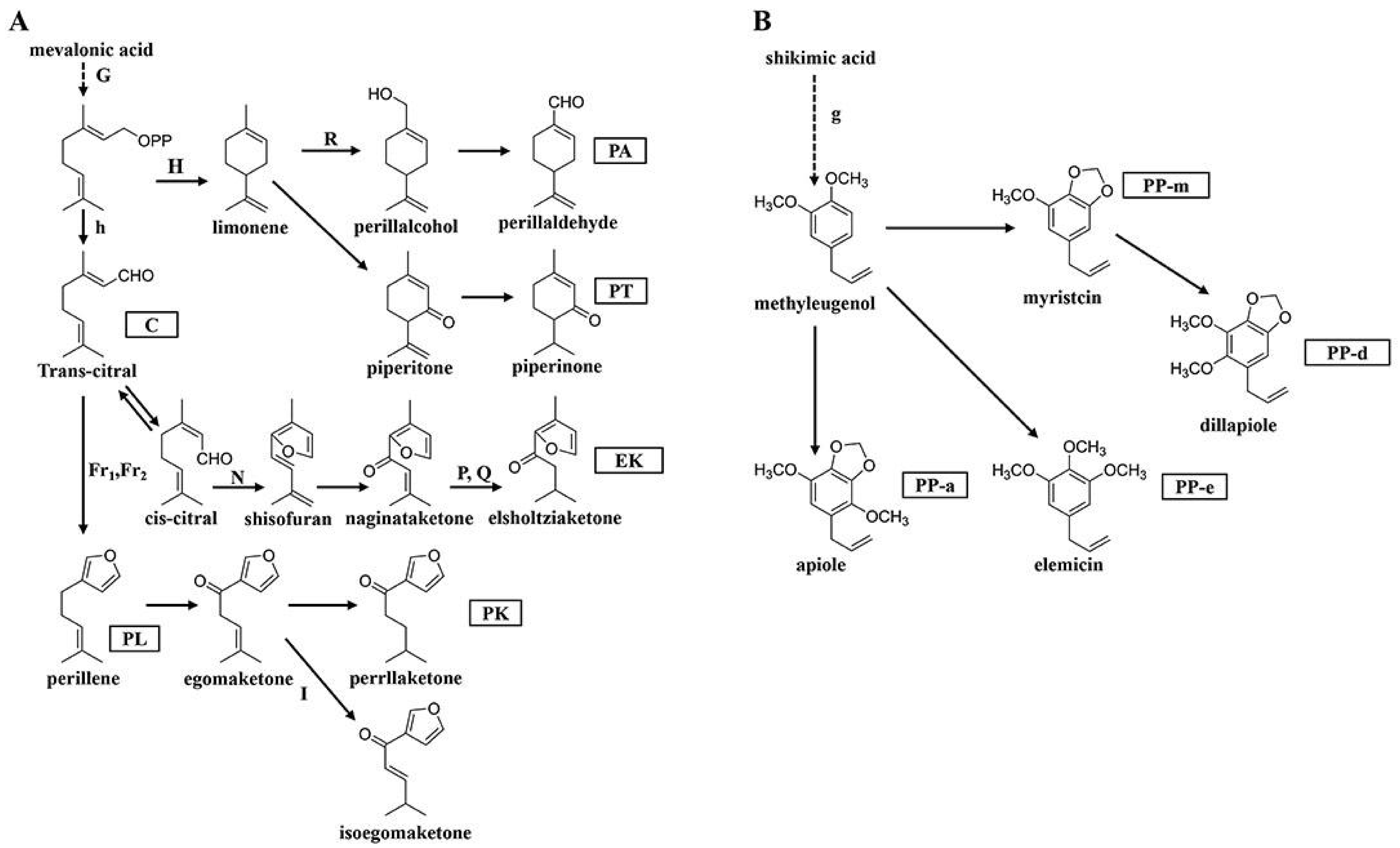

Supplementary Fig.15. Possible reaction steps controlled by genes in hypothetical biosynthetic pathways of chemotypes of *P. frutescens* (Yuba, Honda, Koezuka & Tabata, 1995)**.**
