# Supplementary material for: Multi-omics analysis of the bioactive constituents biosynthesis of glandular trichome in Perilla frutescens
Source: BMC Plant Biol. 2021 Jun 18;21:277. doi: 10.1186/s12870-021-03069-4 (PMC8214284; doi:10.1186/s12870-021-03069-4)
Supplement: Supplementary file 18 — Additional file 18: Methods. [file 12870_2021_3069_MOESM18_ESM.pdf]

Methods

1. RNA extension and transcriptome analysis RNA

1.1 Sample collection and preparation

RNA degradation and contamination was monitored on 1% agarose gels. RNA purity was checked using the Nano Photometer® spectrophotometer (IMPLEN, CA, USA). RNA concentration was measured using Qubit® RNA Assay Kit in Qubit®2.0 Flurometer (Life Technologies, CA, USA). RNA integrity was assessed using the RNA Nano 6000 Assay Kit of the Agilent Bioanalyzer 2100 system (Agilent Technologies, CA, USA).

1.2 cDNA Library preparation for sequencing

A total amount of 2 μg RNA per sample was used as input material for the RNA sample preparations. Sequencing libraries were generated using VAHTSTM mRNA-seq V2 Library Prep Kit for Illumina® following manufacturer's recommendations and index codes were added to attribute sequences to each sample. Briefly, mRNA was purified from total RNA using poly-T oligo-attached magnetic beads. Fragmentation was carried out using divalent cations under elevated temperature in VAHTSTM First Strand Synthesis Reaction Buffer（5X）. First strand cDNA was synthesized using random hexamer primer and M-MuLV Reverse Transcriptase（RNase H-）. Second strand cDNA synthesis was subsequently performed using DNA polymerase I and RNase H. Remaining overhangs were converted into blunt ends via exonuclease/polymerase activities. After adenylation of 3' ends of DNA fragments, Adaptor were ligated to prepare for library. In order to select cDNA fragments of preferentially 150~200 bp in length, the library fragments were purified with AMPure XP system (Beckman Coulter, Beverly, USA). Then 3 μl USER Enzyme (NEB, USA) was used with size-selected, adaptor-ligated cDNA at 37°C for 15 min followed by 5 min at 95 °C before PCR. Then PCR was performed with Phusion High-Fidelity DNA polymerase, Universal PCR primers and Index (X) Primer. At last, PCR products were purified (AMPure XP system) and library quality was assessed on the Agilent Bioanalyzer 2100 system. The libraries were then quantified and pooled. Paired-end sequencing of the library was performed on the HiSeq XTen sequencers (Illumina, San Diego, CA).

1.3 Data assessment and quality control

FastQC (version 0.11.2) was used for evaluating the quality of sequenced data. Raw data was filtered by **Trimmomatic** (version 0.36) according to several steps: 1) Removing sequences with N bases; 2) Removing adaptor sequence if reads contains; 3) Removing low quality bases from reads 3' to 5' ($Q < 20$); 4) Removing low quality bases from reads 5' to 3' ($Q < 20$); 5) Using a sliding window method to remove the base value less than 20 of reads tail (window size is 5 bp); 6) Removing reads with reads length less than 35nt and its pairing reads. And the remaining clean data was used for further analysis.

1.4 Transcriptome assembly and gene annotation

The remaining clean reads were de novo assembled into transcripts using **Trinity** (version 2.0.6) with default settings. Transcripts with a minimum length of 200 bp were clustered to minimize redundancy. For each cluster (representing the transcriptional complexity for the same gene), the longest sequence was preserved and designated as unigene. Unigenes were blasted against NCBI Nr (NCBI non-redundant protein database), SwissProt, TrEMBL, CDD (Conserved Domain Database), Pfam and KOG (eukaryotic Orthologous Groups) databases (E-value < 1e-5). According to the priority order of the best aligned results of NR, SwissProt and TrEMBL to determine the Unigene ORF, and then determining its CDS and the corresponding amino acid sequences according to the codon table. At the same time, **TransDecoder** (version 3.0.1) was used to predict CDS sequences of the un-aligned Unigenes. GO (Gene Ontology database) functional annotation information was obtained according to transcripts annotation results of SwissProt and TrEMBL. **KAAS (**version 2.1) (KEGG Automatic Annotation Server) was used for KEGG (Kyoto Encyclopedia of Genes and Genomes) annotation.

1.5 RNA-seq assessment

Bowtie2 (version 2.3.2) was used for aligning the quality control sequences to the assembled transcripts, and **RSeQC** (version 2.6.1) was used for statistics the aligned result. Then **BEDTools (**version 2.26.0) was used for homogeneity distribution check and statistics the gene coverage ratio, **RSeQC** (version 2.6.1) software was used for duplicate reads analysis.

1.6 Expression quantity analysis

The direct expression of a gene expression level is the abundance of its transcript; higher transcript abundance represents higher gene expression level. Transcripts Per Million (TPM) is a measure to calculate the proportion of a transcript in the RNA pool. It takes into account the sequence depth and the length of the gene as well as the influence of the sample on the reads count. **Salmon (**version 0.8.2) was used to calculate the reads count and TPM of unigenes. Differentially expressed genes were calculated based on the reads count of each gene.

For the samples without biological repetition, TMM was used to standardize the read count data, and then **DEGseq** (version 1.26.0) was used for differently analysis. For the samples with biological repetition, **DESeq** (version 1.12.4) was used for analysis. In order to obtain the significant differential genes, the screening conditions were set as follows: q-value <0.001 and difference multiple |FoldChange| >2.

1.7 Relationship analysis of samples

The gene expression correlation between samples is an important index to test whether the experiment is reliable and the sample selection is reasonable. The closer the correlation coefficient gets to 1, the more similarity the expression patterns between samples.

Principal Component Analysis (PCA) could reflect the distance and difference between samples through different sample species and function composition analysis, and the related graph was constructed by **R vegan** package.

Principal co-ordinates analysis (PCoA) is a visualization method to study the similarity or difference of data, and the difference between individuals or groups can be observed by PCoA. The related graph was constructed by **R vegan** package.

Non-metric multidimensional scale (NMDS) is used to map, analyze and classify the research objects (samples or variables) of the multidimensional space into the low-dimensional space, while preserving the original relationship between the objects. The related graph was performed by **R vegan** package.

1.8 Functional enrichment analysis

TopGO (version 2.24.0) was used for GO (Gene Ontology) enrichment, and the function was thought to be a significant enrichment when the correct p-value (q-value) < 0.05. The basic unit of GO is GO-term. GO enrichment analysis provides all GO terms that significantly enriched in DEGs comparing to the genome background.

ClusterProfiler (version 3.0.5) was used for Kyoto Encyclopedia of Genes and Genomes (KEGG) enrichment analysis. Pathway enrichment analysis identified significantly enriched metabolic pathways or signal transduction pathways in DEGs comparing with the whole genome background.

Protein-protein interaction network was performed with **R igraph** package. STRING is a protein interaction database developed by EMBL, which has collected the most powerful experimental verification, data mining and homologous prediction of protein interactions. The differentially expressed genes were mapped to the responding protein-protein interaction network to extract sub network and make it visualization, screening key genes according to the topology of genes in the whole network.
